# Supplementary material for: Serine 25 phosphorylation inhibits RIPK1 kinase-dependent cell death in models of infection and inflammation
Source: Nat Commun. 2019 Apr 15;10:1729. doi: 10.1038/s41467-019-09690-0 (PMC6465317; doi:10.1038/s41467-019-09690-0)
Supplement: Supplementary file 1 — Supplementary Information [file 41467_2019_9690_MOESM1_ESM.pdf]

Serine 25 phosphorylation inhibits RIPK1 kinase-dependent cell death in models of infection and inflammation

Dondelinger, Delanghe *et al.*

Supplementary Figure 1

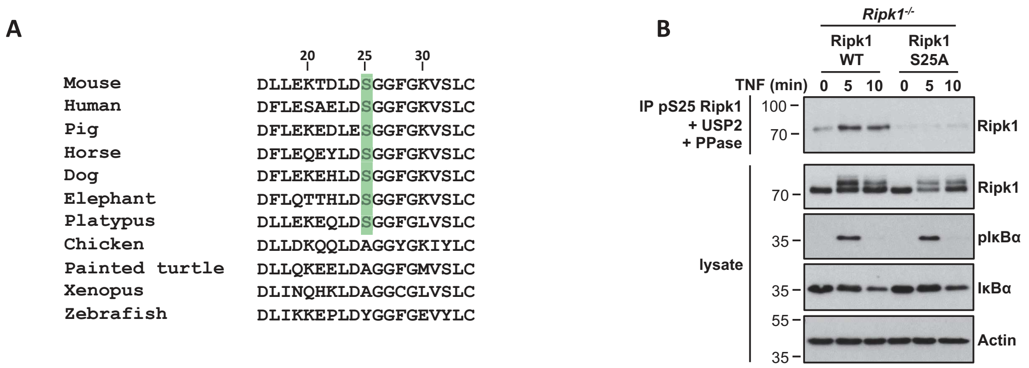

Supplementary Figure 1. Evolutionary conservation of Serine 25 and validation of the pS25 antibody. (**A**) Overview of Ser25 conservation (green box) in RIPK1 from selected species. (**B**) *Ripk1*$^{-/-}$ MEFs lentivirally reconstituted with wild-type (WT) or the S25A RIPK1 mutant were stimulated with 1μg/ml hTNF for the indicated duration. pS25 RIPK1 was then immunoprecipitated and treated with USP2 and λ phosphatase (PPase) post-IP. Protein levels were determined by immunoblot. Immunoblots are representative of 1 independent experiment.

Supplementary Figure 2

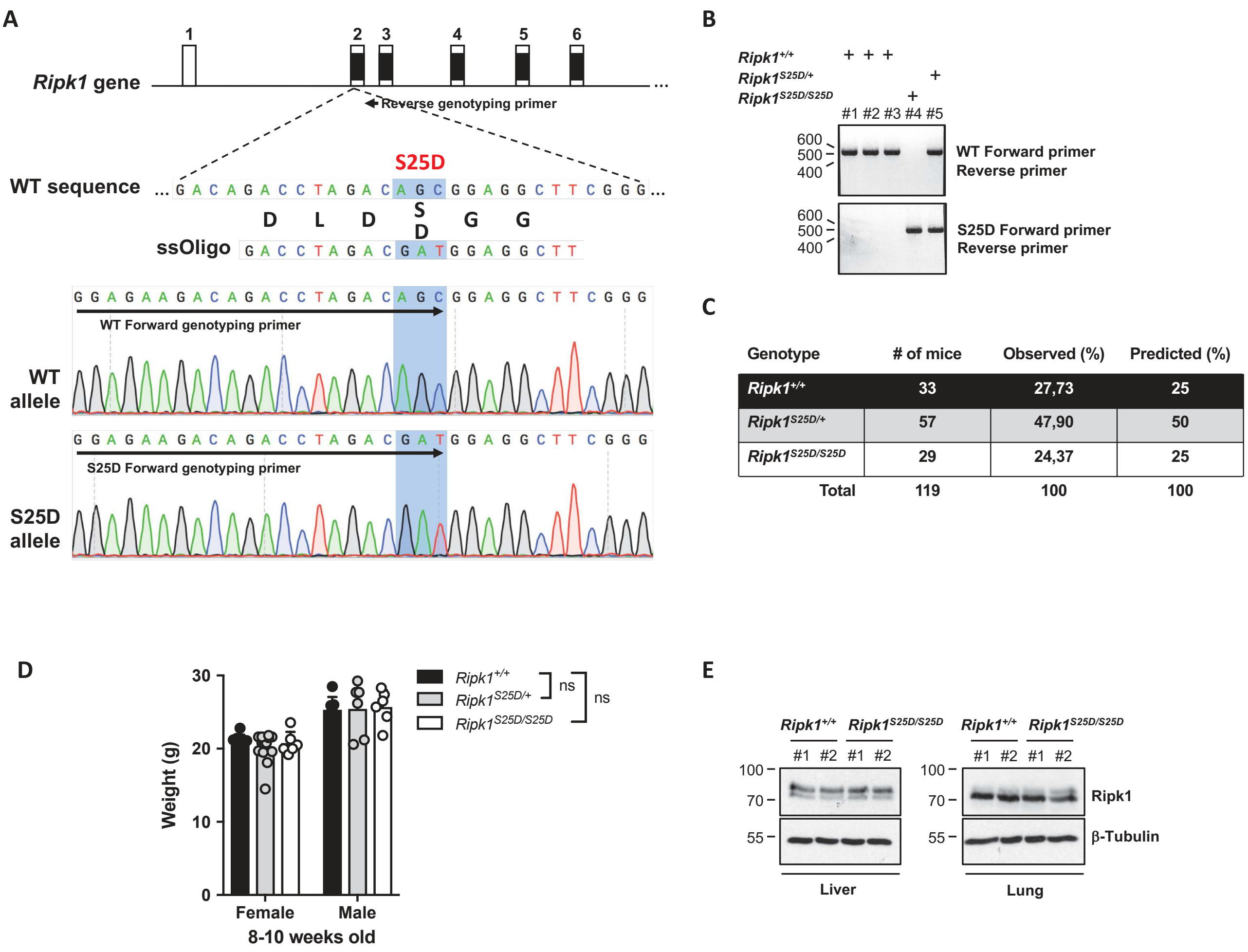

Supplementary Figure 2. Generation and general phenotyping of the *Ripk1*$^{S25D/S25D}$ mouse line. (**A**) The *Ripk1*$^{S25D/S25D}$ mouse line was generated by altering AGC serine-encoding codon in exon 2 of the mouse RIPK1 locus to a GAT codon encoding for glutamate by using the CRISPR-cas9 technology. DNA sequencing was used to confirm mutagenesis from AGC to GAT. (**B**) Genotyping strategy to identify the presence of the S25D mutation. (**C**) Observed and predicted Mendelian frequencies of the RIPK1 alleles after *Ripk1*$^{S25D/+}$ heterozygous crosses. (**D**) Mice of the indicated genotypes were weighed at 8-10 weeks of age. Weight measurements are presented as mean ± SEM from n different mice. Statistical significance was determined by two-way ANOVA followed by a Tukey post hoc test. (*Ripk1*$^{+/+}$ female n=10, male n=7; *Ripk1*$^{S25D/+}$ female n=13, male n=6; *Ripk1*$^{S25D/S25D}$ female n=6, male n=6). ns: non-significant. (**E**) Littermate adult mice from *Ripk1*$^{S25D/+}$ heterozygous crosses were sacrificed and lung and liver protein levels were determined by immunoblot. Immunoblots are representative of 2 (**B**,**E**) independent experiments.

Supplementary Figure 3

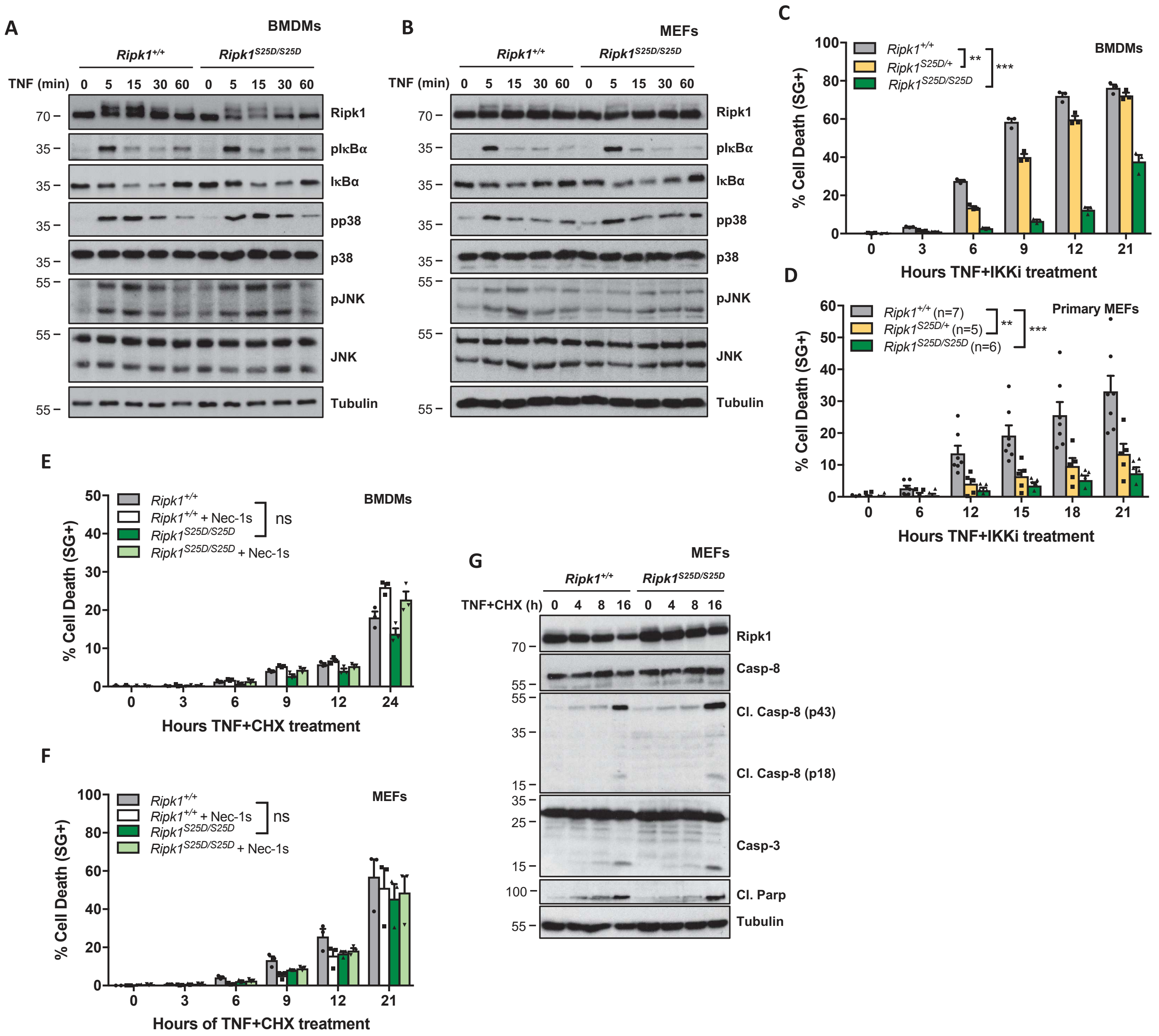

Supplementary Figure 3. Mimicking Ser25 phosphorylation does not affect TNF signaling to MAPK/NF-kB but specifically protects from TNF-induced RIPK1 kinase-dependent cell death. (**A**-**G**) *Ripk1*$^{+/+}$, *Ripk1*$^{S25D/+}$ and *Ripk1*$^{S25D/S25D}$ BMDMs (**A**, **C**, **E**) or MEFs (**B**, **D**, **F**-**G**) were pretreated with the indicated compounds for 30min before stimulation with hTNF (1ng/ml for BMDMs and 20ng/ml for MEFs). Activation of cytosolic proteins was monitored by immunoblotting (**A**, **B**, **G**) and cell death was measured in function of time by SytoxGreen positivity (**C**-**F**). Cell death data are presented as mean ± SEM of at least 3 independent experiments (**D**, n indicated in figure; **F**, n=3). Results were obtained with BMDMs isolated from 3 different mice for each genotype (n=3) (**C**, **E**). Statistical significance for the cell death assays was determined using two-way ANOVA followed by a Tukey post-hoc test. Significance between samples is indicated in the figures as follows: ∗∗: p< 0.01; ∗∗∗: p< 0.001; ns: non-significant. Immunoblots are representative of 2 (**A**-**B**,**G**) independent experiments.

Supplementary Figure 4

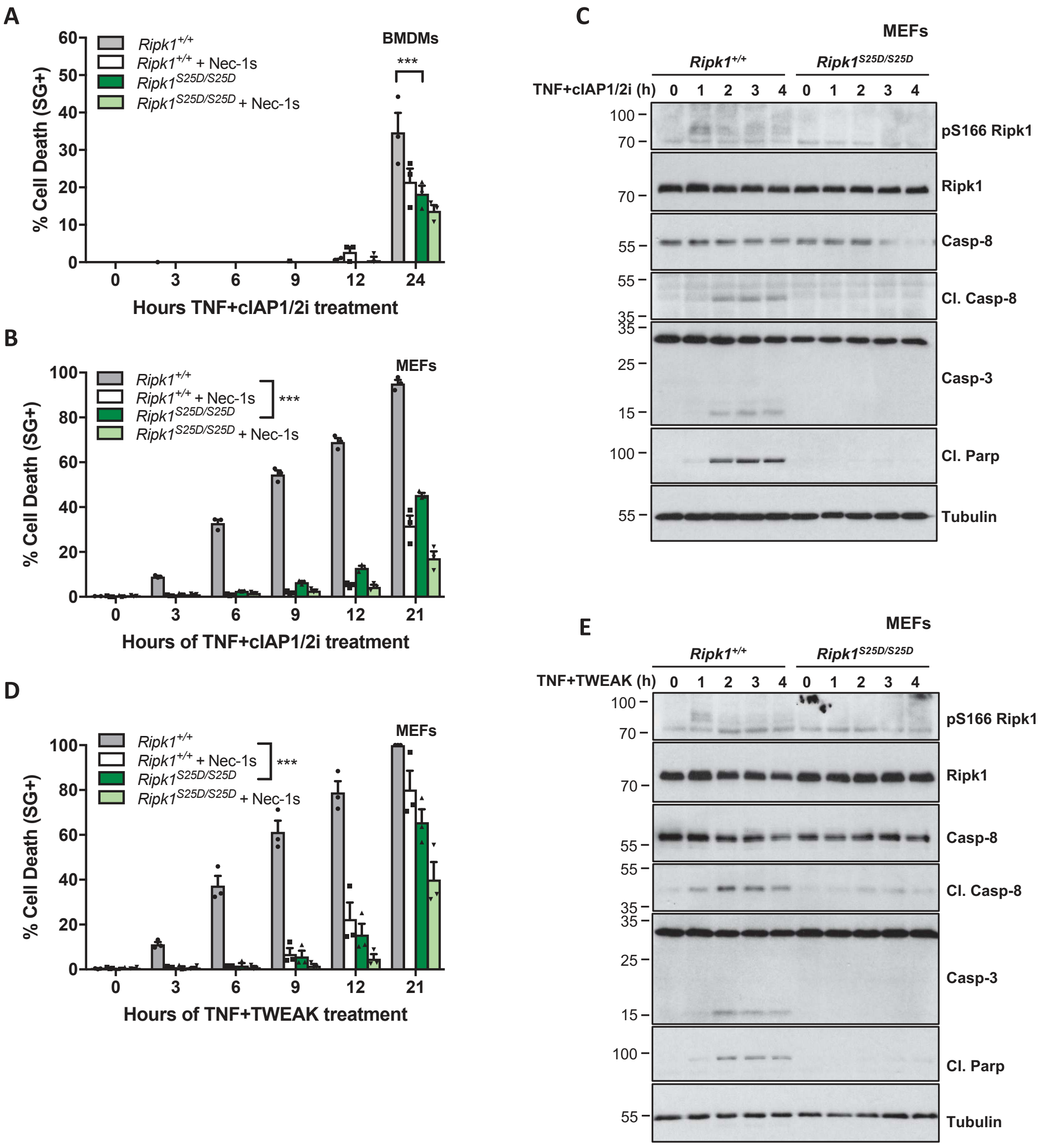

Supplementary Figure 4. Mimicking Ser25 phosphorylation protects cells from TNF-induced RIPK1 kinase-dependent death in presence of cIAP1/2 inhibitor or TWEAK co-stimulation. (**A**-**E**) *Ripk1*$^{+/+}$ and *Ripk1*$^{S25D/S25D}$ BMDMs (**A**) or MEFs (**B**-**E**) were pretreated for 2h with TWEAK (100 ng/ml) or cIAP1/2i (BV6) prior to hTNF stimulation (10ng/ml for BMDMs and 20ng/ml for MEFs). Cell death (**A**, **B**, **D**) and protein activation (**C**, **E**) were measured in function of time respectively by SytoxGreen positivity and immunoblotting. Cell death data are presented as mean ± SEM of 3 independent experiments (n=3). BMDMs were isolated from 3 different mice for both genotypes (n=3). Statistical significance for the cell death assays was determined using two-way ANOVA followed by a Tukey post-hoc test. Significance between samples is indicated in the figures as follows: ∗∗∗: p< 0.001. Immunoblots are representative of 2 (**C**,**E**) independent experiments.

Supplementary Figure 5

Figure 1B

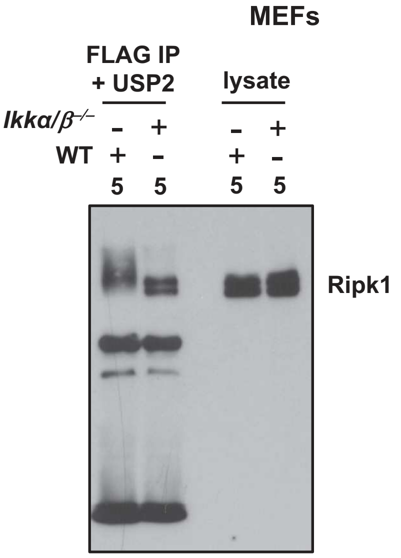

Figure 1D

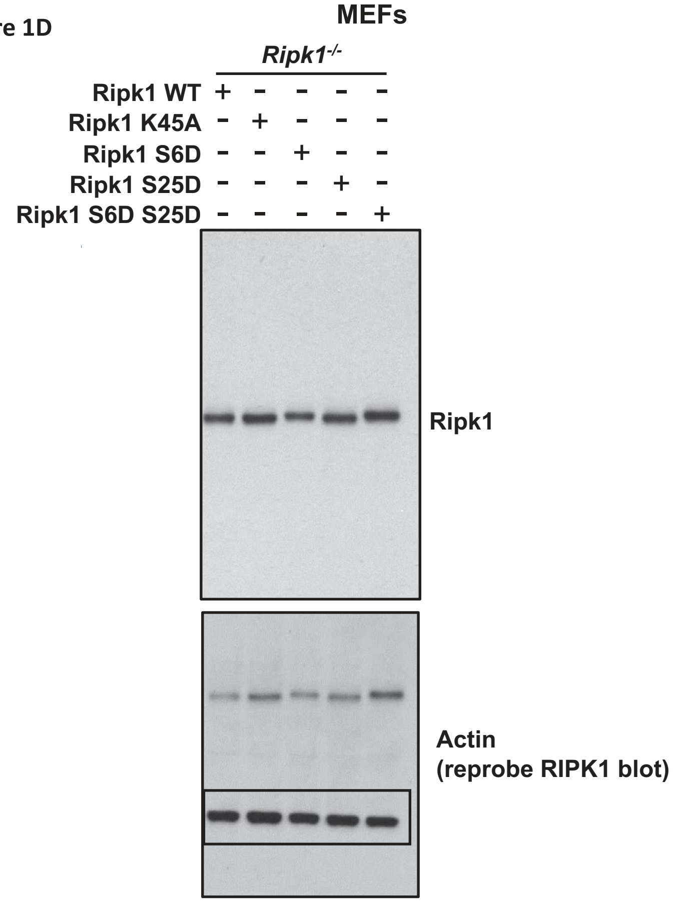

Figure 1E

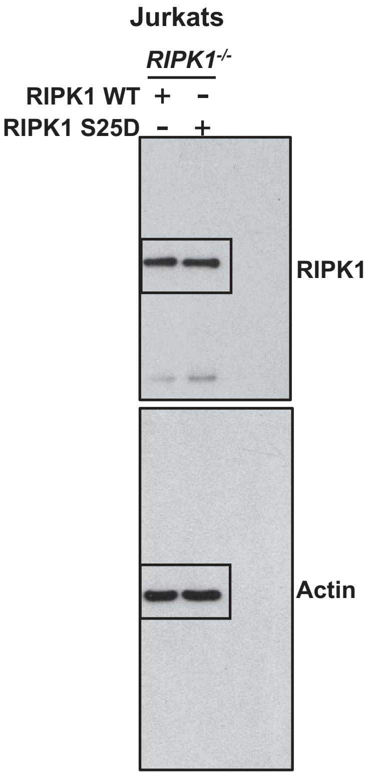

Supplementary Figure 5. Western blot source data. Uncropped versions of all the western blots presented in the manuscript

Supplementary Figure 5: Source data immunoblots (continued)

Figure 1F

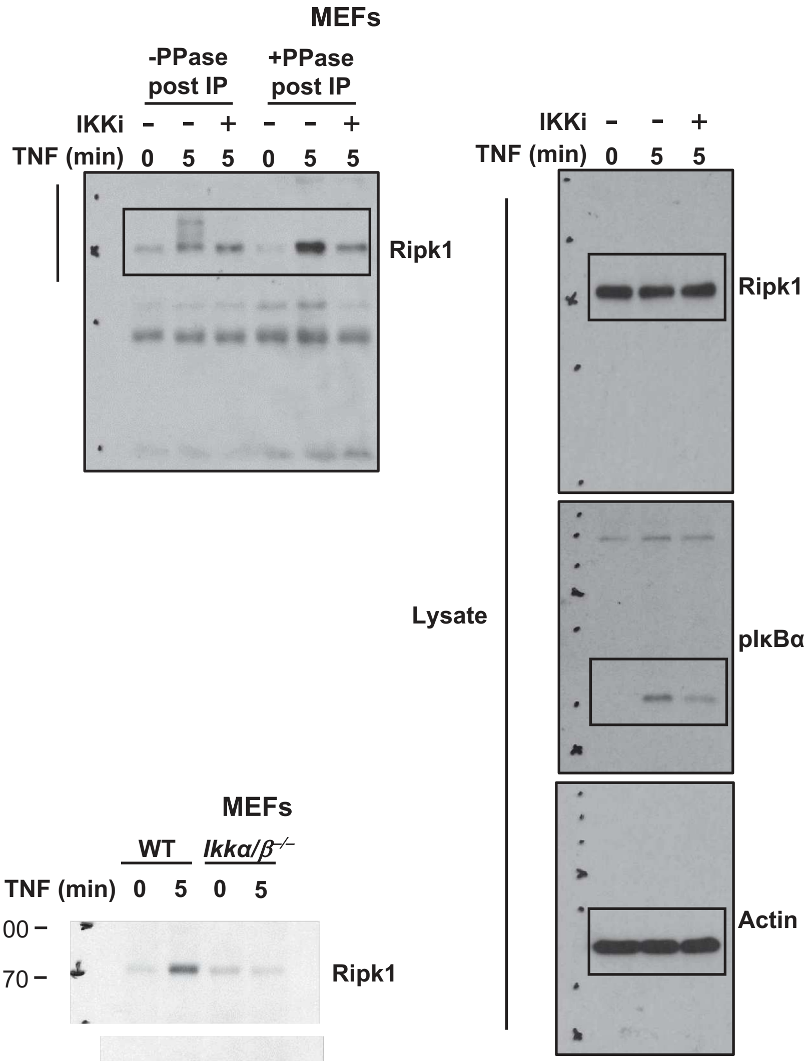

Figure 1G

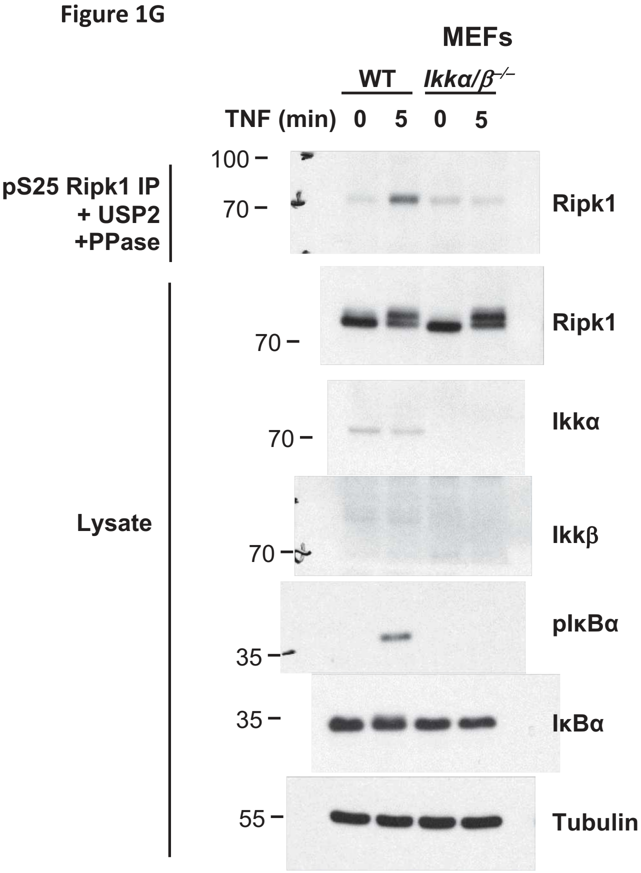

Supplementary Figure 5: Source data immunoblots (continued)

Figure 1H

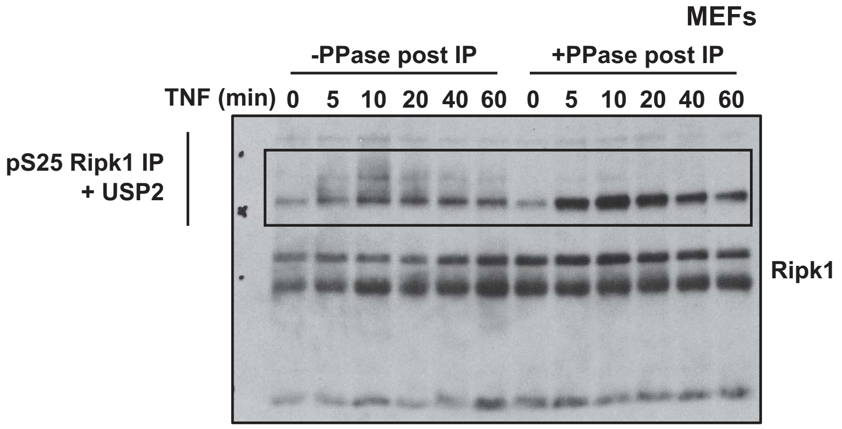

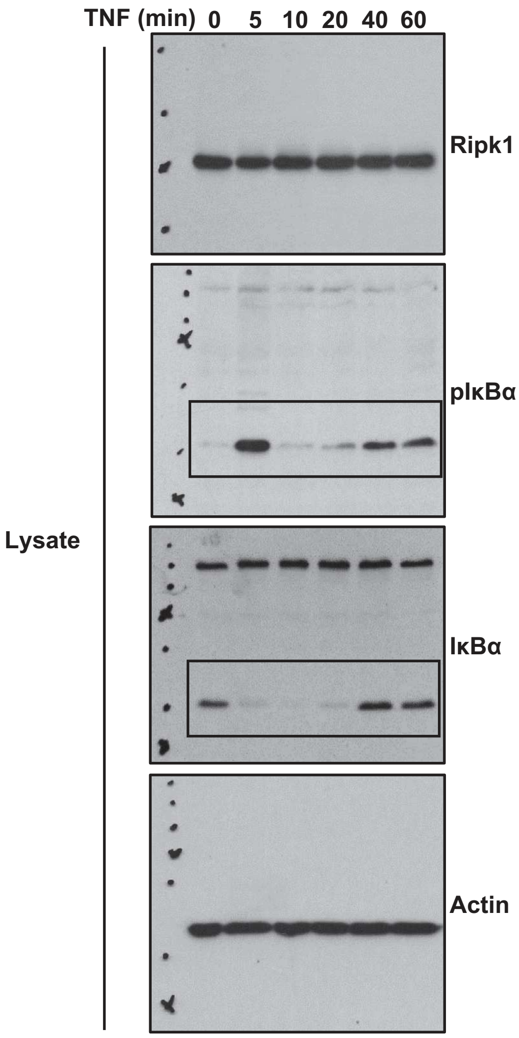

Supplementary Figure 5: Source data immunoblots (continued)

Figure 1I

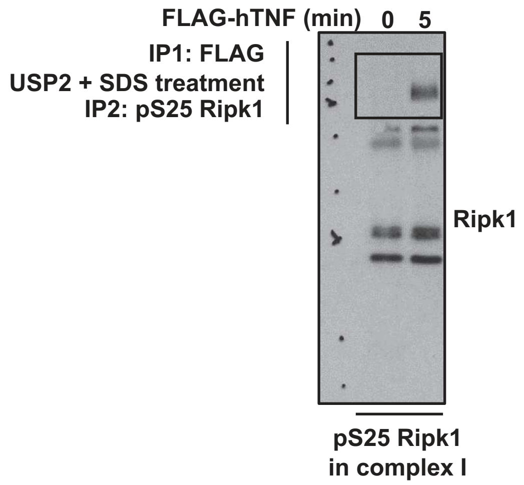

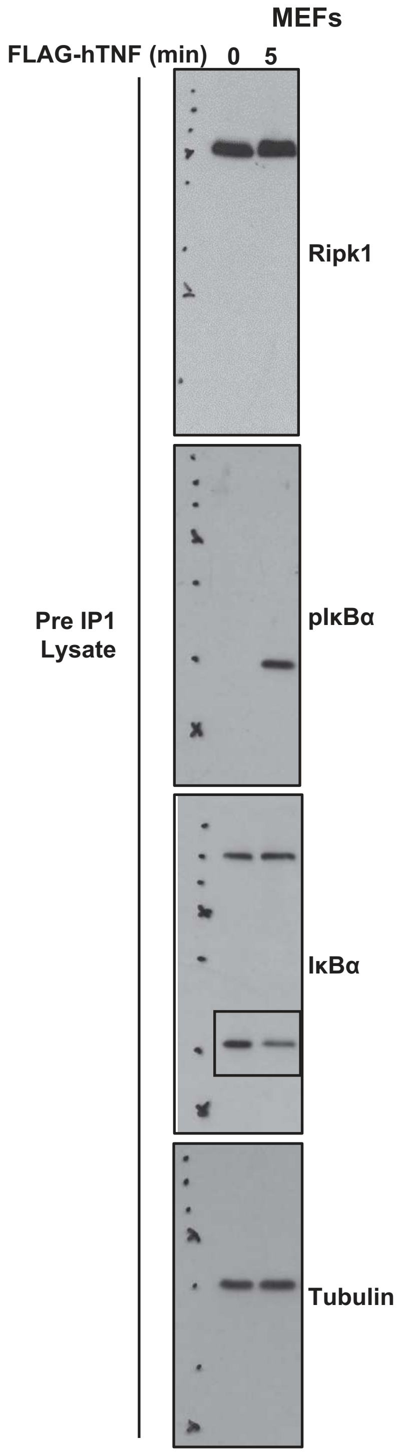

Figure 1J

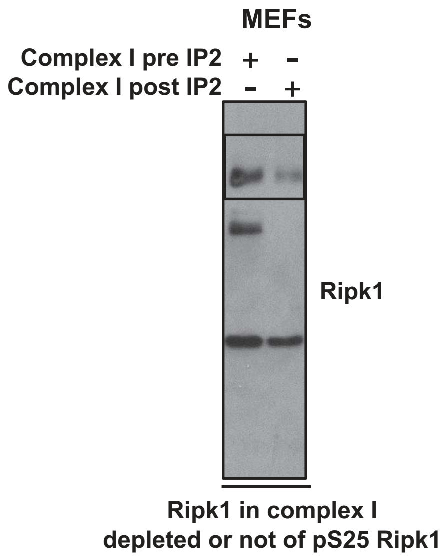

Supplementary Figure 5: Source data immunoblots (continued)

Figure 1K

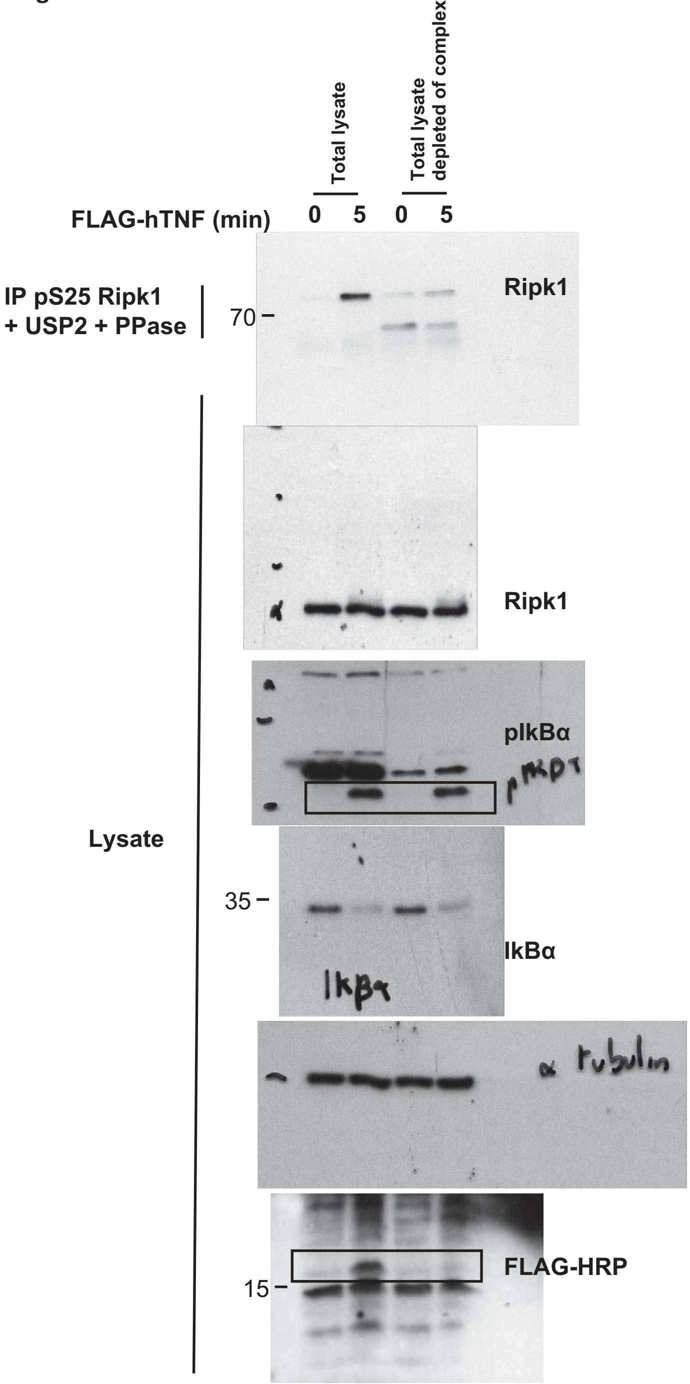

Figure 1L

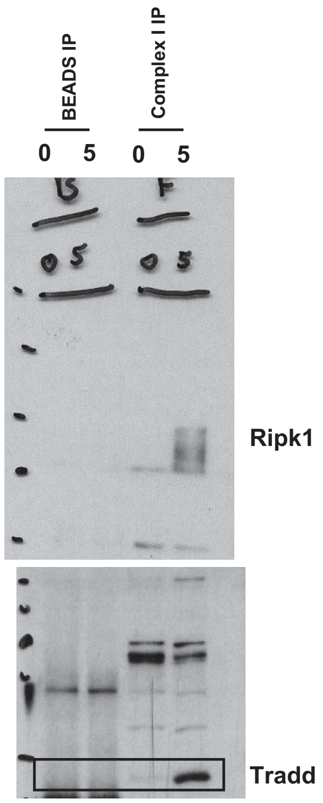

Supplementary Figure 5: Source data immunoblots (continued)

Figure 2C

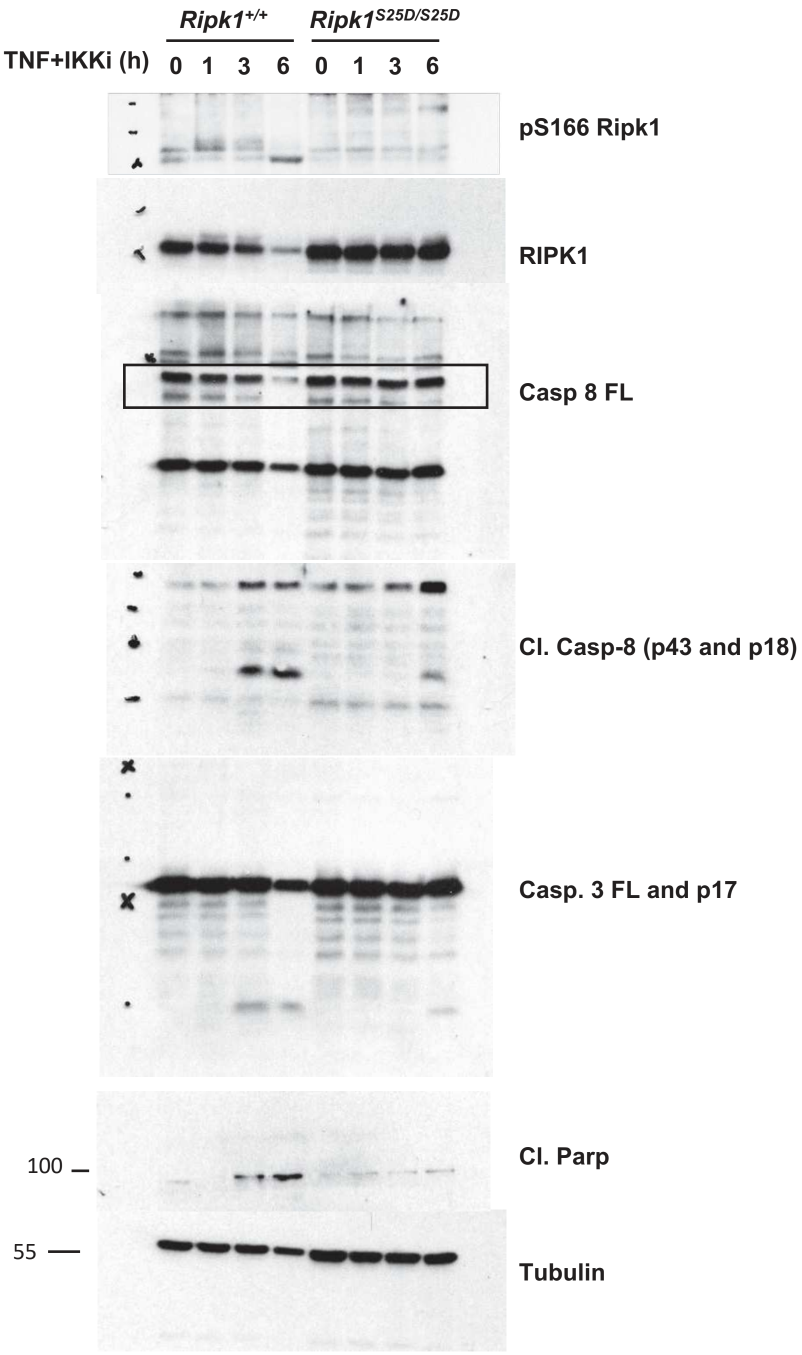

Supplementary Figure 5: Source data immunoblots (continued)

Figure 2F

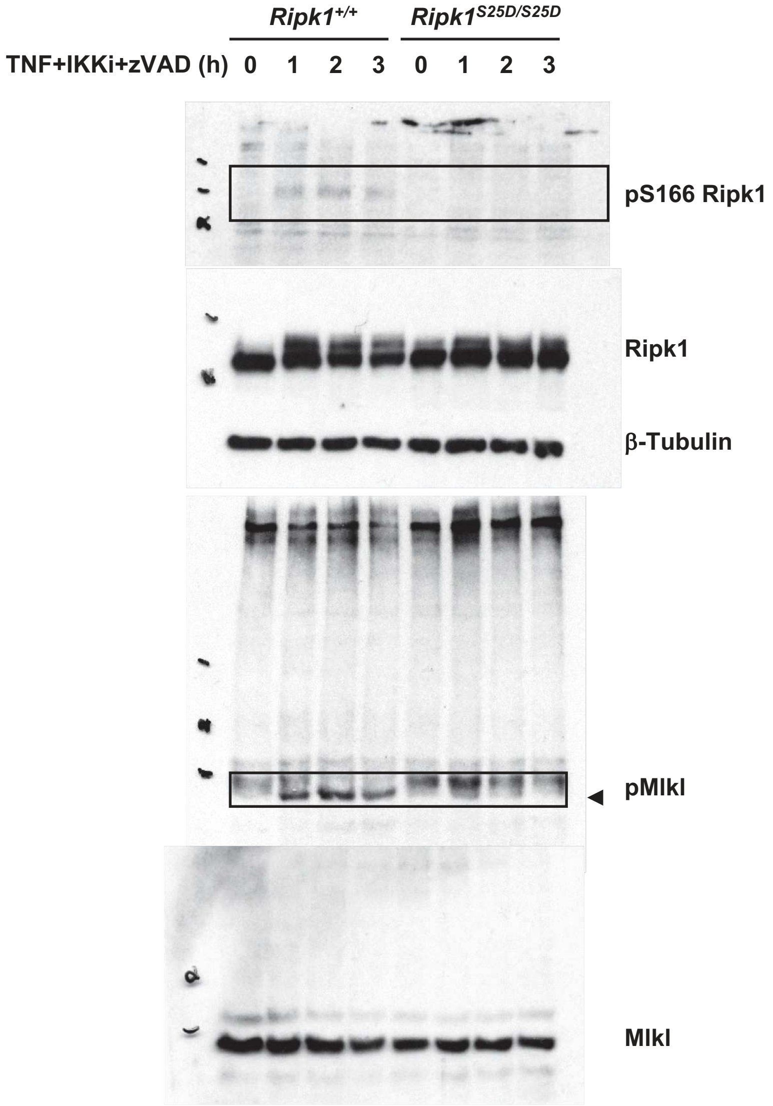

Supplementary Figure 5: Source data immunoblots (continued)

Figure 2G

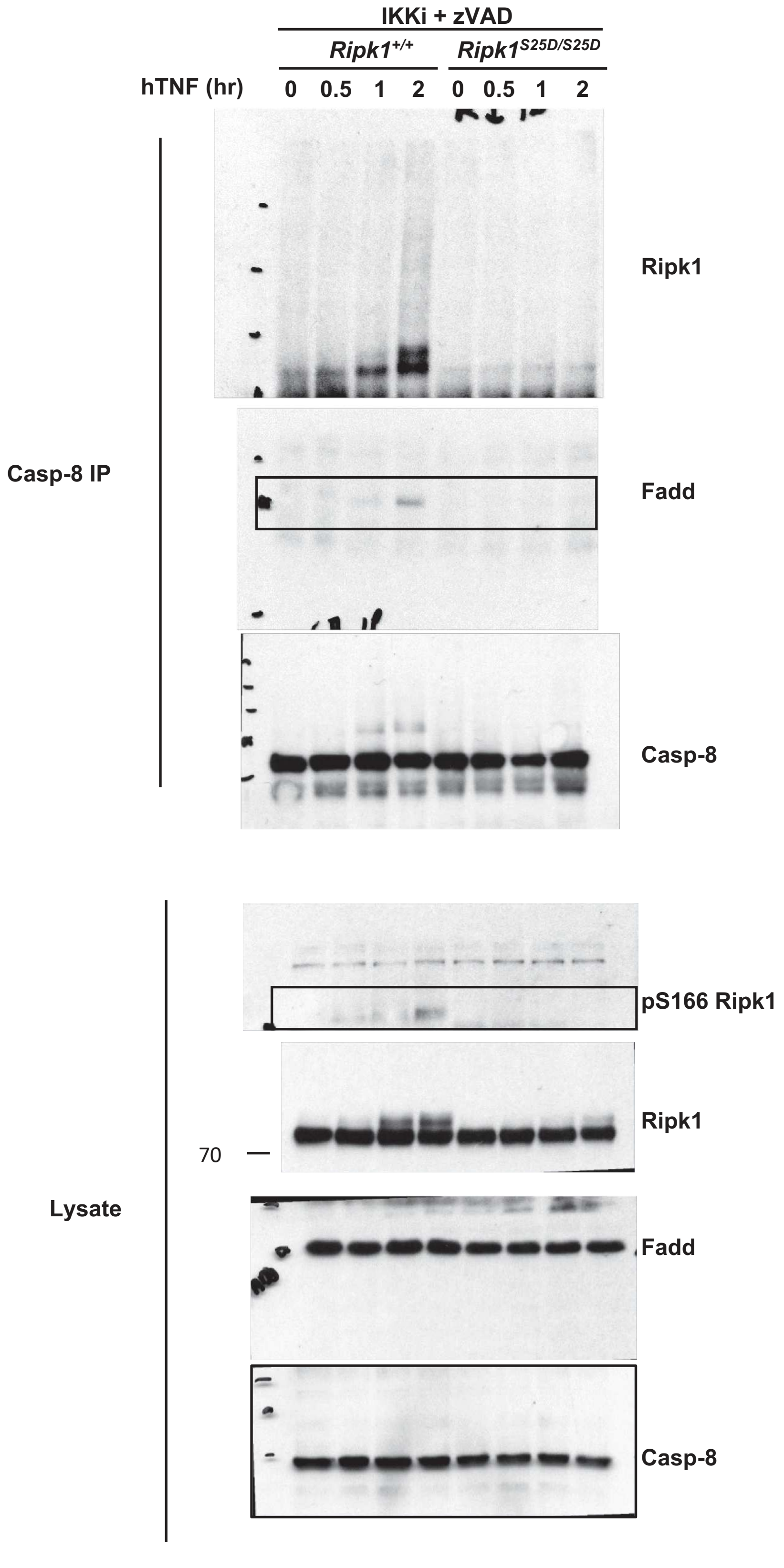

Supplementary Figure 5: Source data immunoblots (continued)

Figure 3A

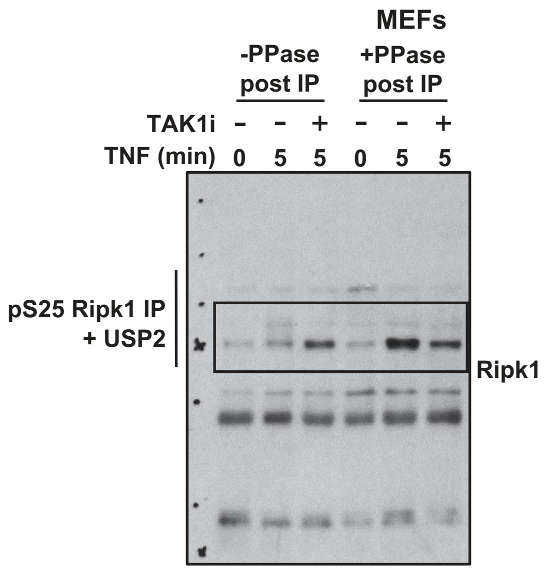

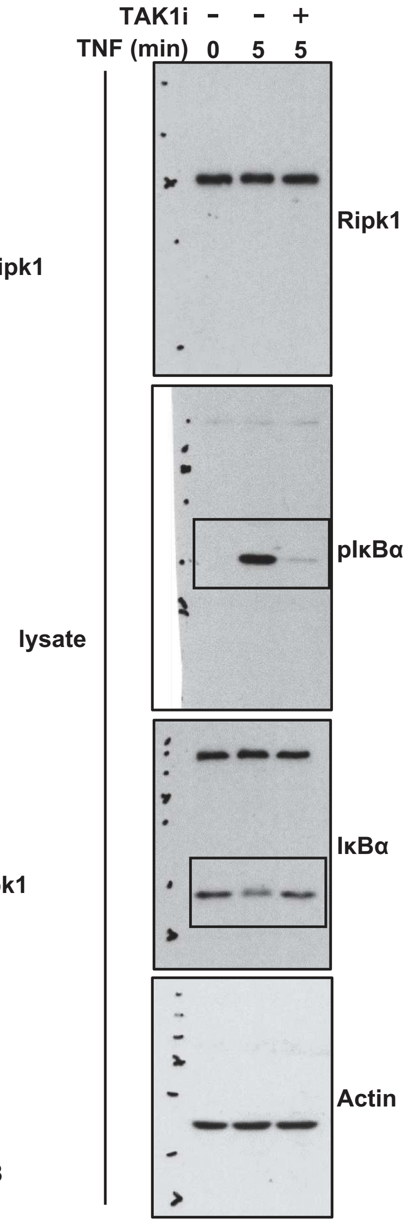

Figure 3D

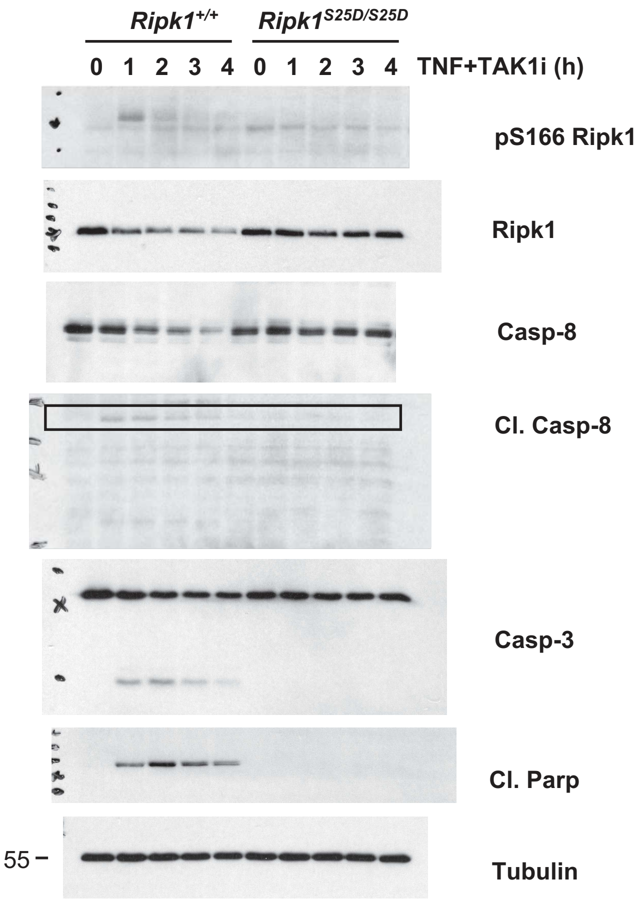

Supplementary Figure 5: Source data immunoblots (continued)

Figure 3I

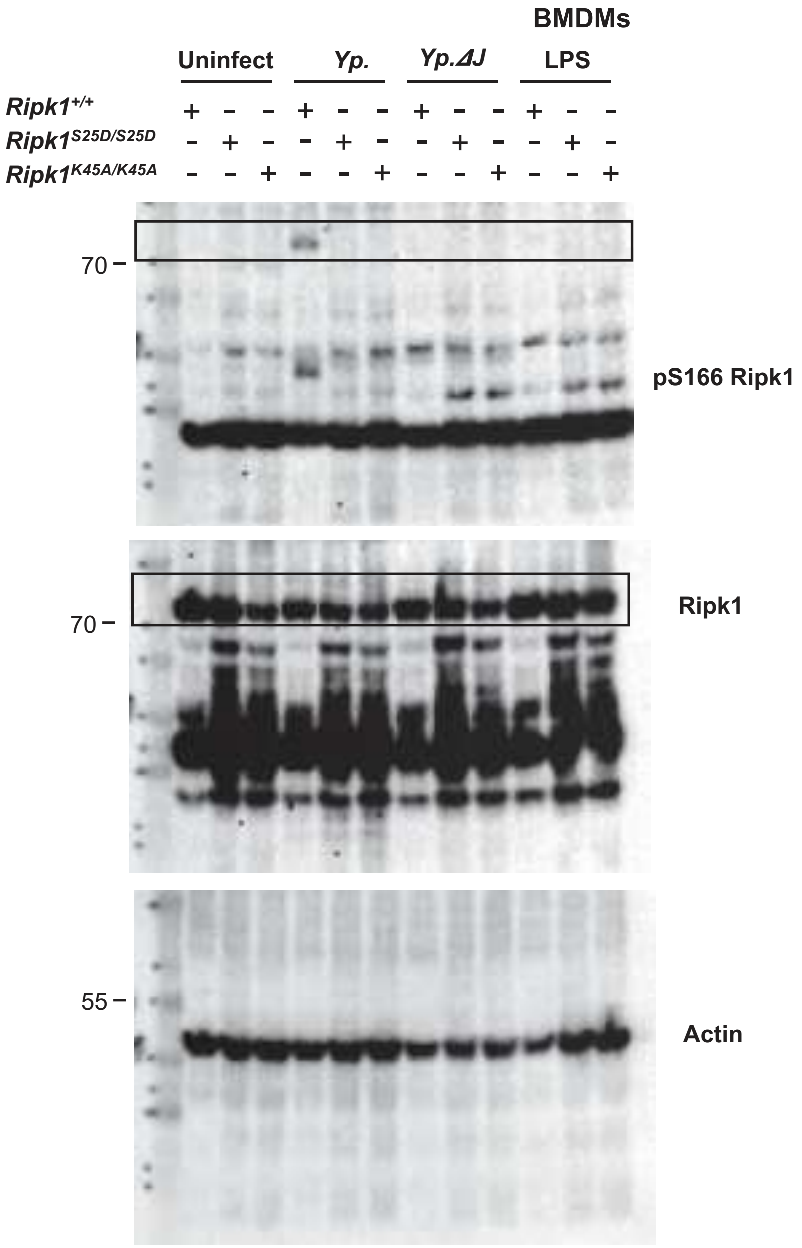

Supplementary Figure 5: Source data immunoblots (continued)

Figure 4A

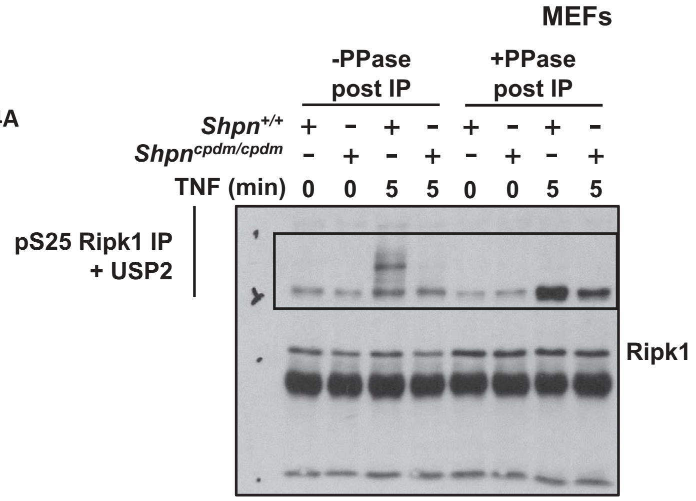

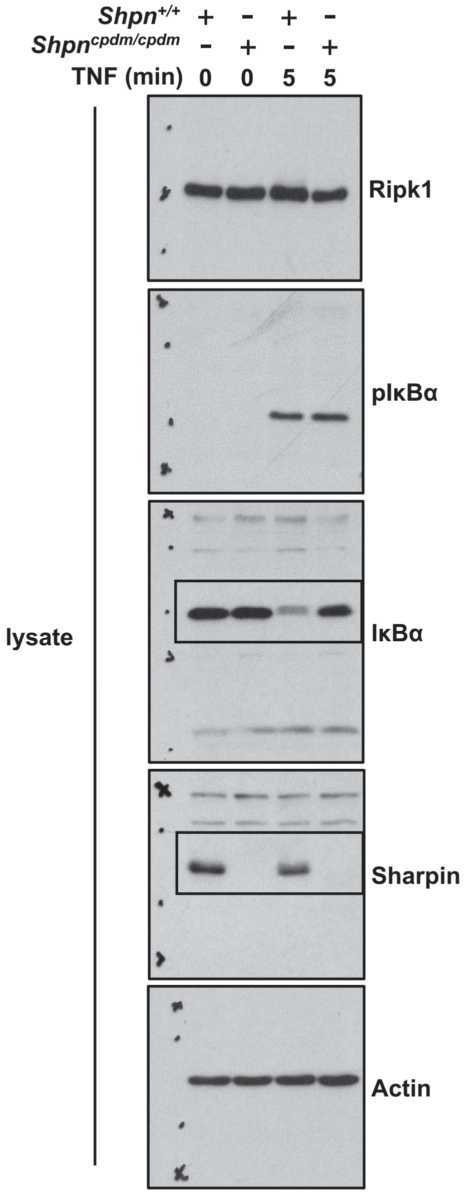

Figure 4C

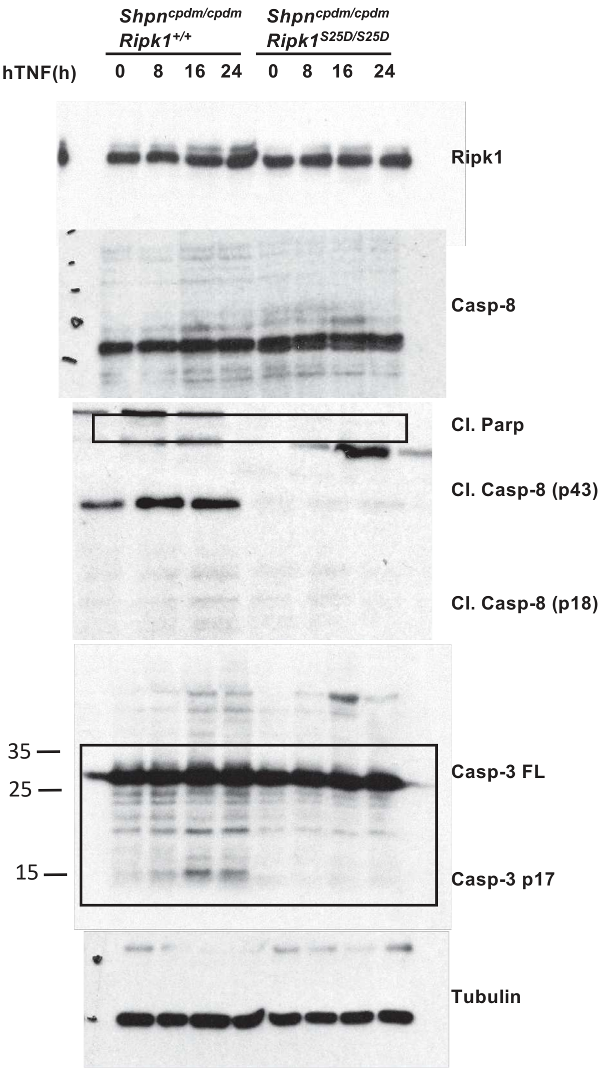

Supplementary Figure 5: Source data immunoblots (continued)

Figure 5A

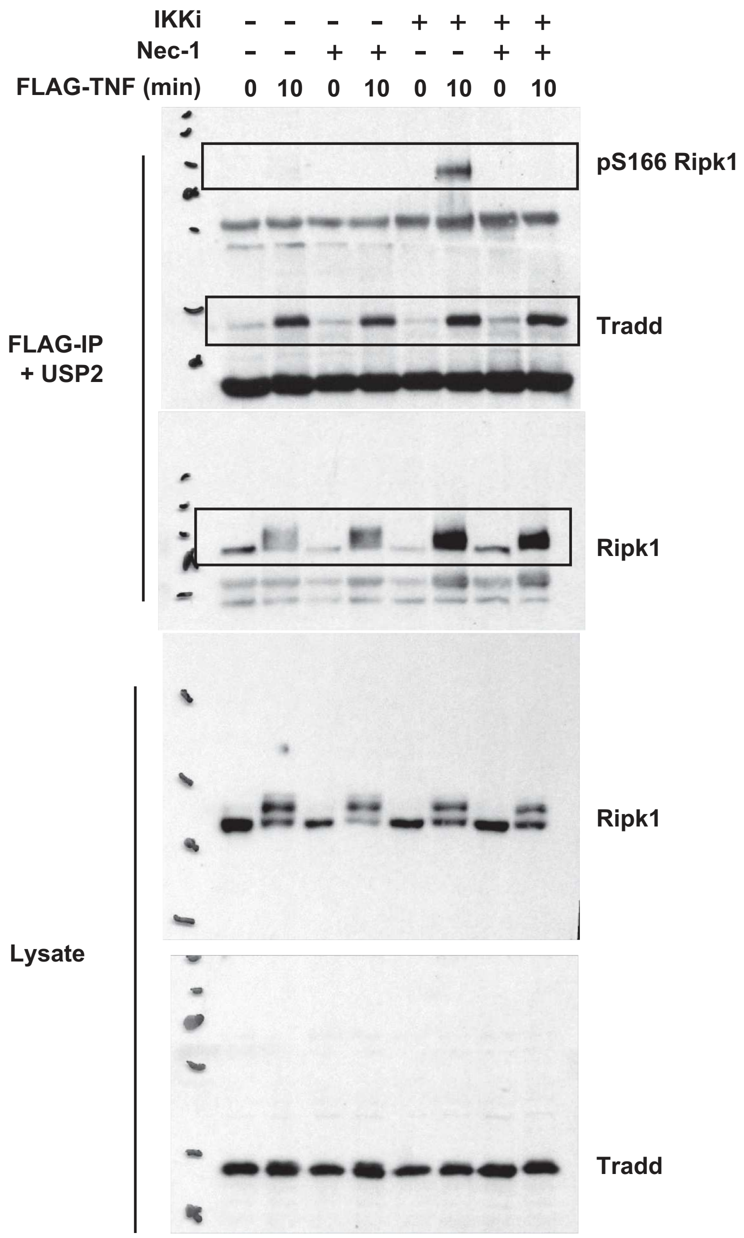

Supplementary Figure 5: Source data immunoblots (continued)

Figure 5B

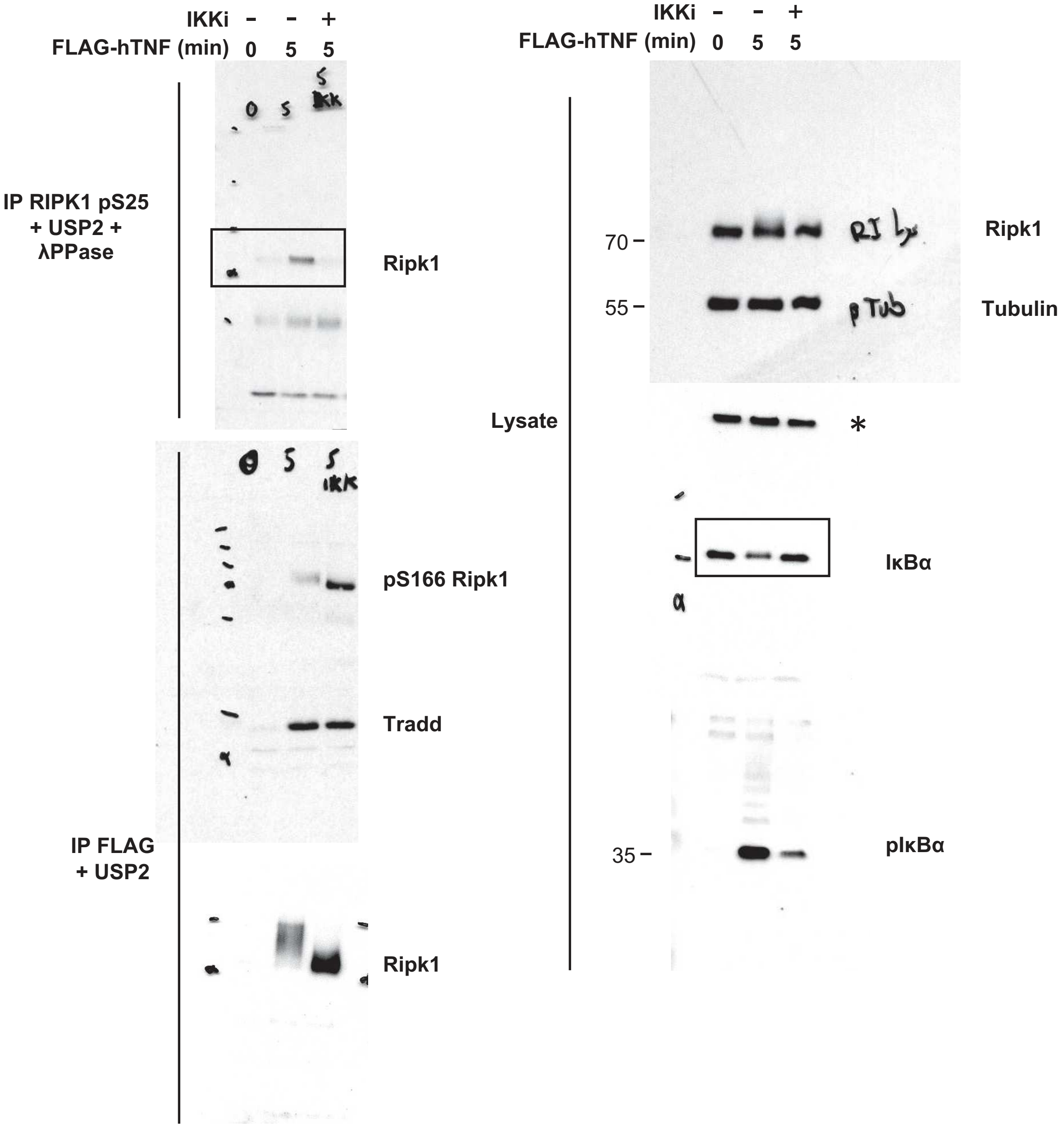

Supplementary Figure 5: Source data immunoblots (continued)

Figure 5C

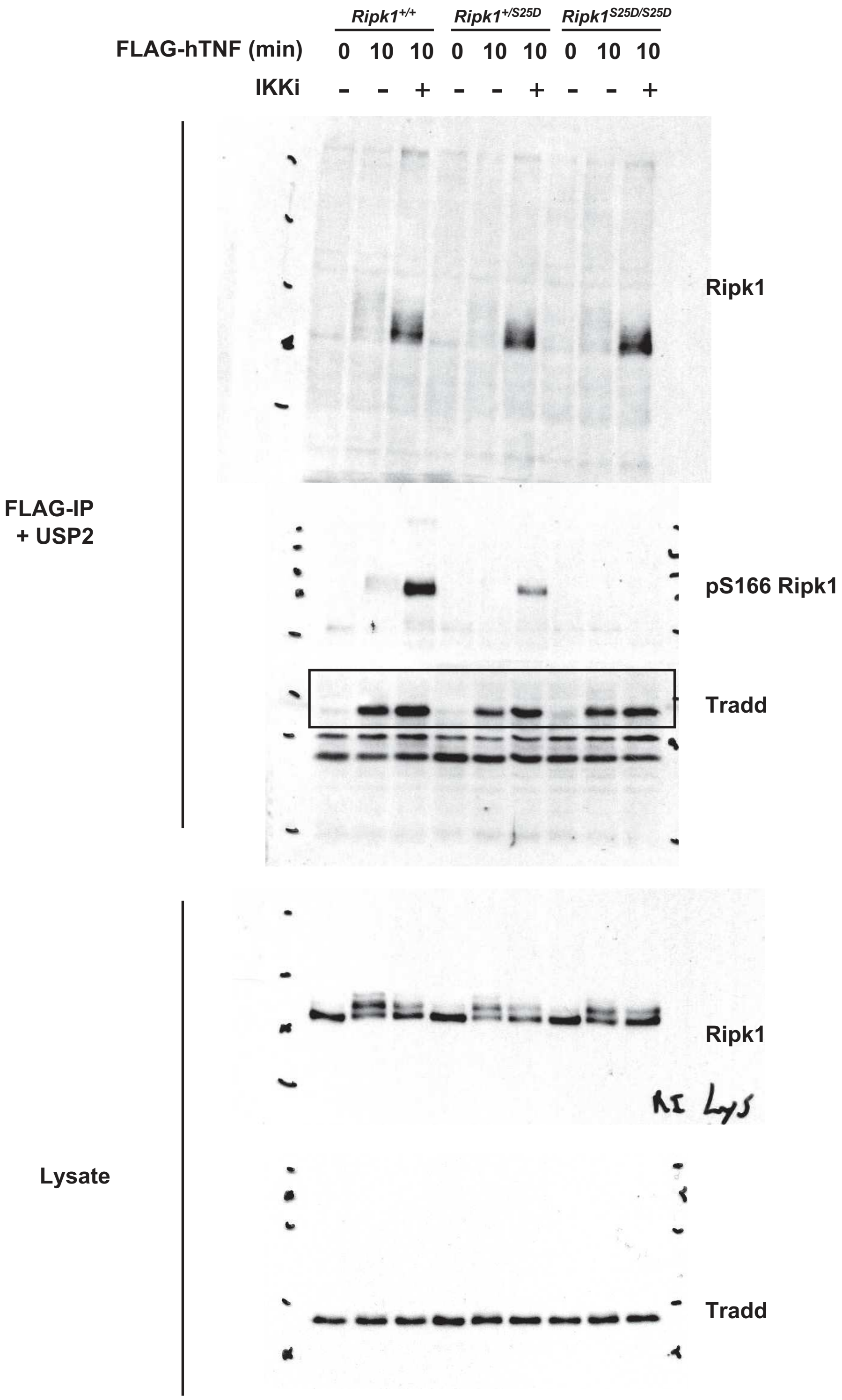

Supplementary Figure 5: Source data immunoblots (continued)

Figure 5D

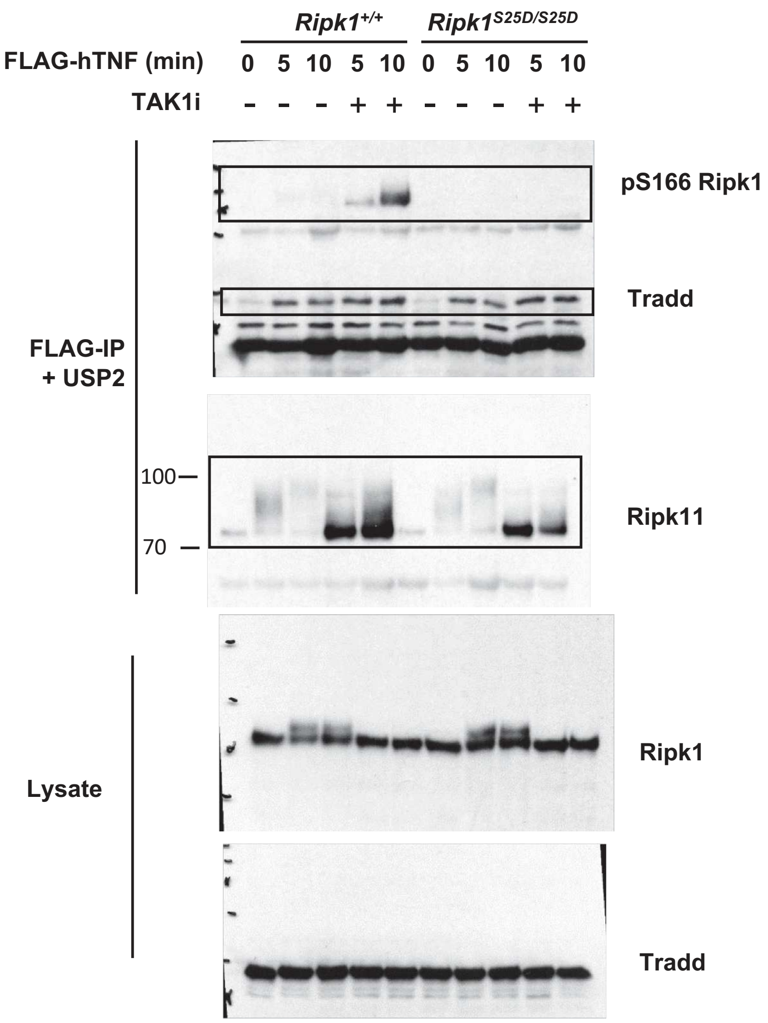

Supplementary Figure 5: Source data immunoblots (continued)

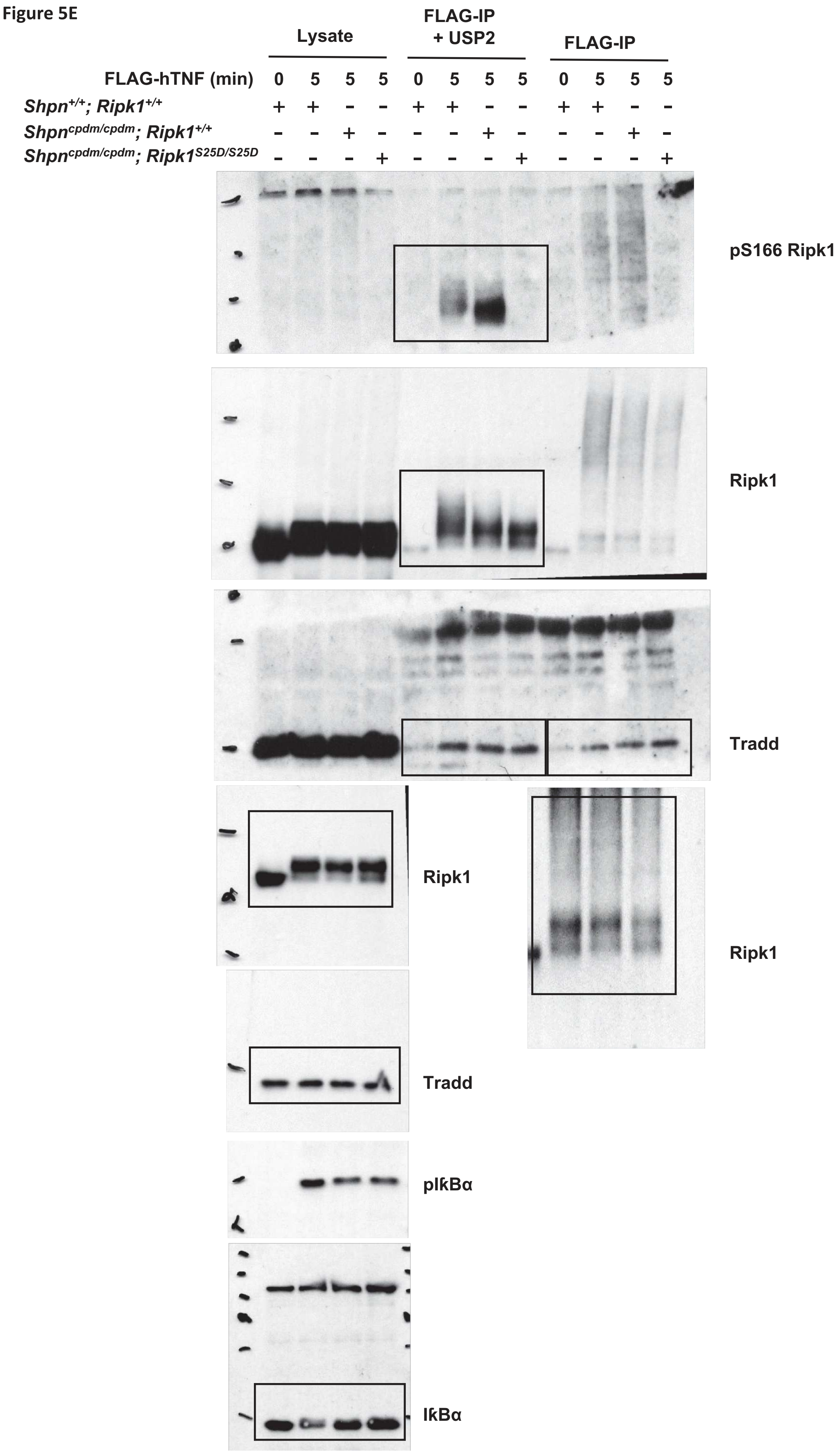

Supplementary Figure 5: Source data immunoblots (continued)

Figure 5F

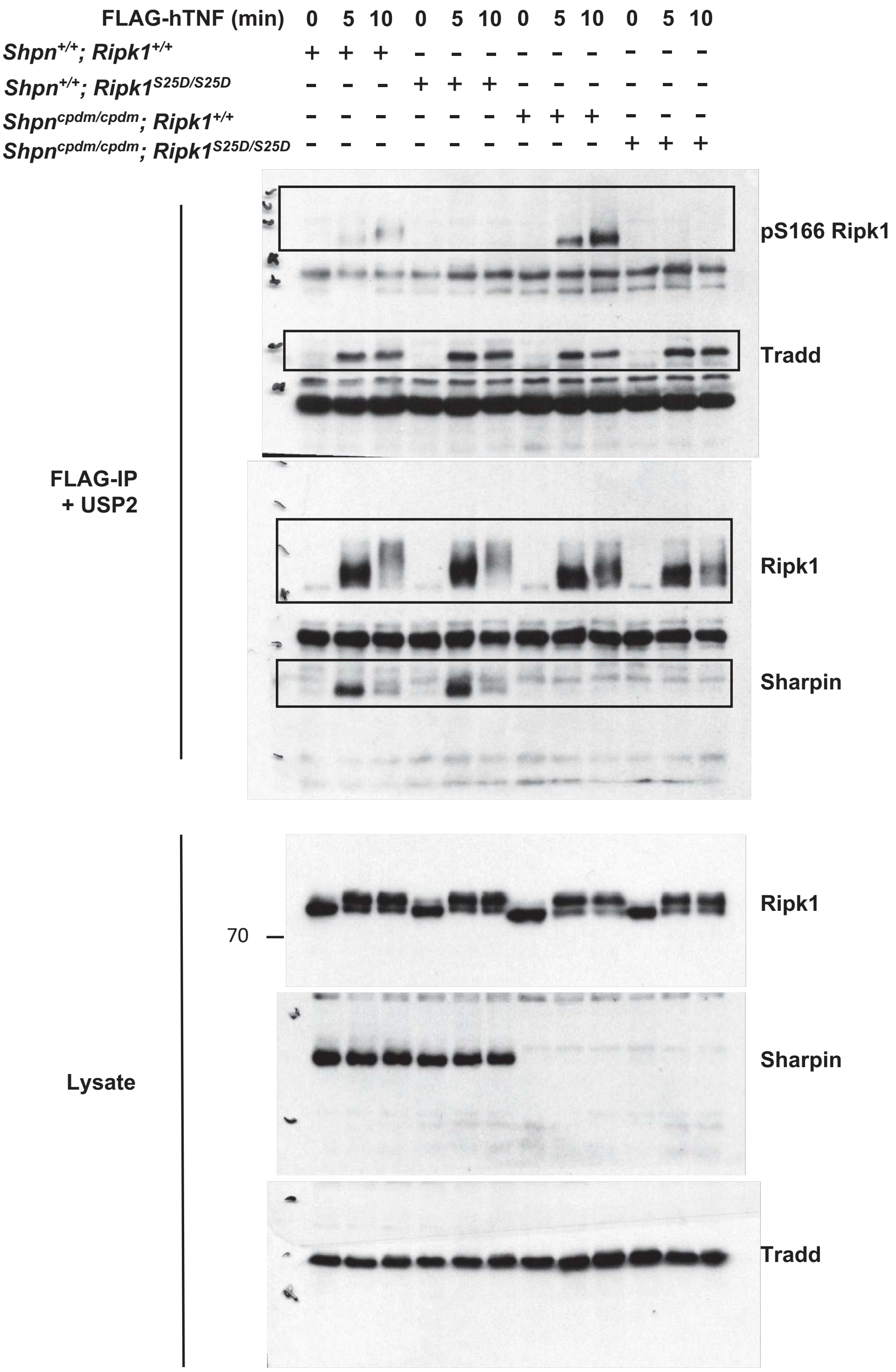

Supplementary Figure 5: Source data immunoblots (continued)

Figure 6E

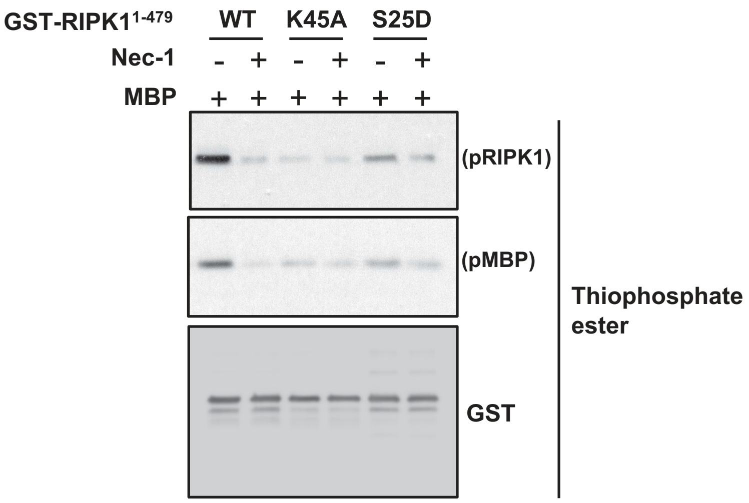

Figure 7A

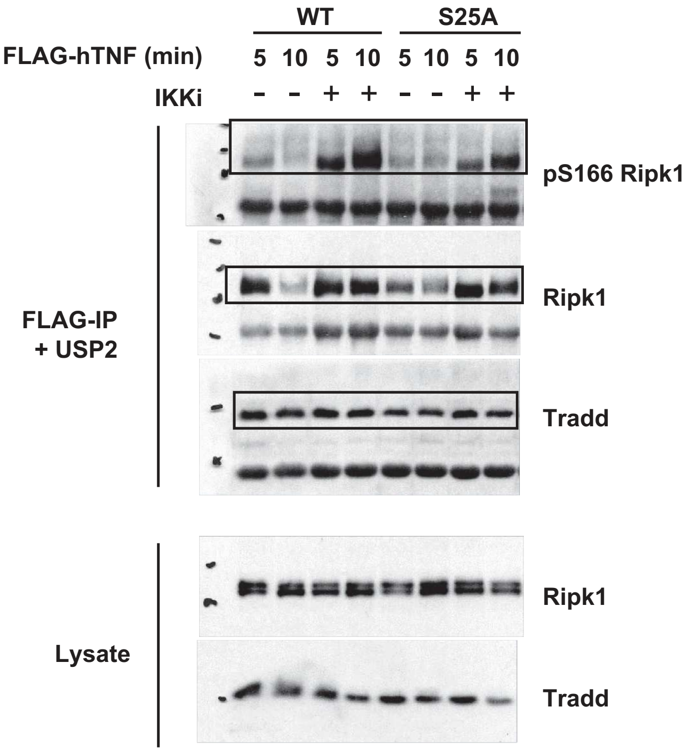

Supplementary Figure 5: Source data immunoblots (continued)

Suppl. Figure 1B

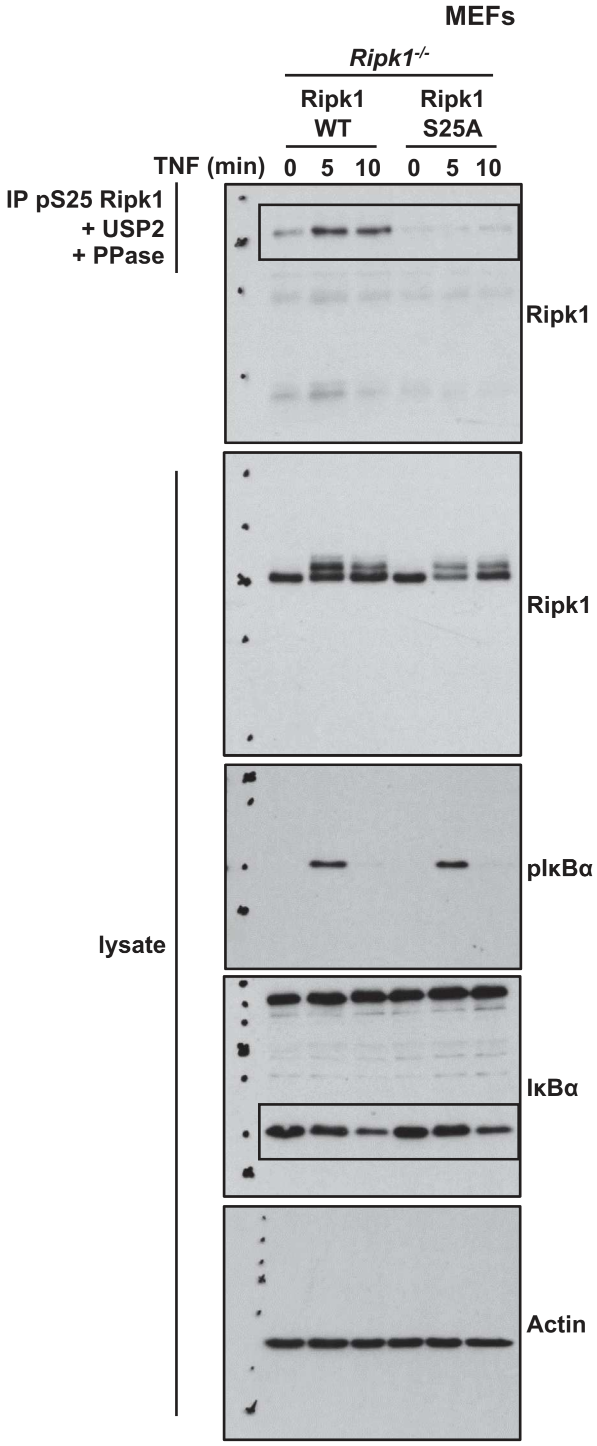

Suppl. Figure 2E

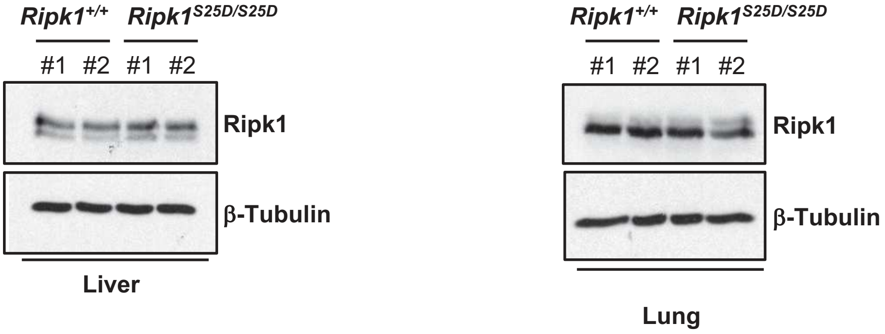

Supplementary Figure 5: Source data immunoblots (continued)

Suppl. Figure 3A

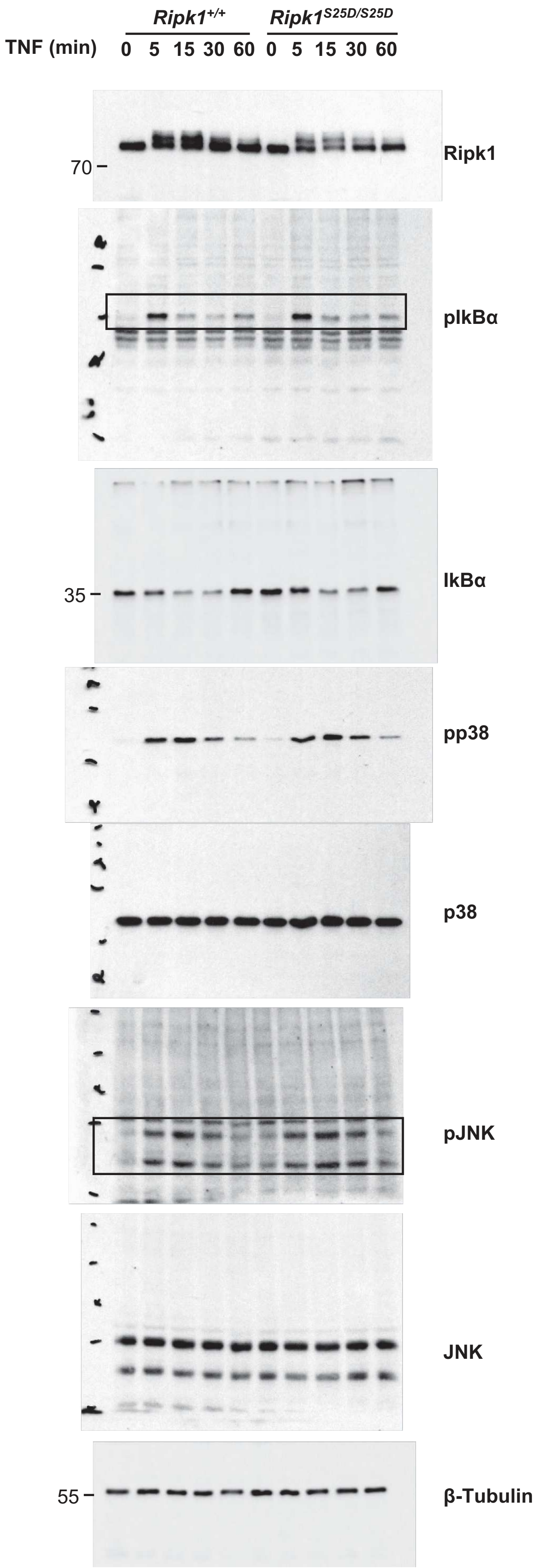

Supplementary Figure 5: Source data immunoblots (continued)

Suppl. Figure 3B

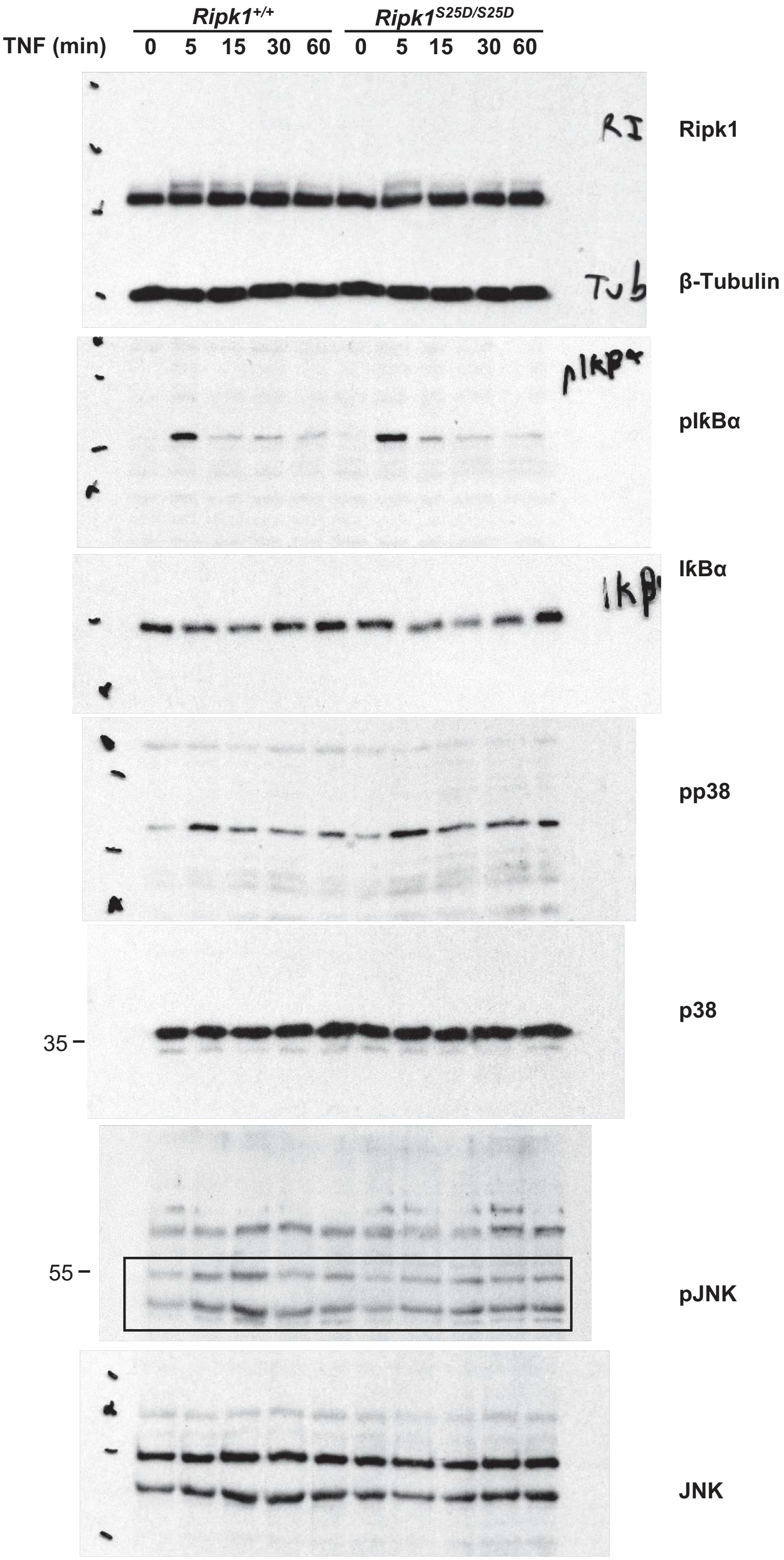

Supplementary Figure 5: Source data immunoblots (continued)

Suppl. Figure 3G

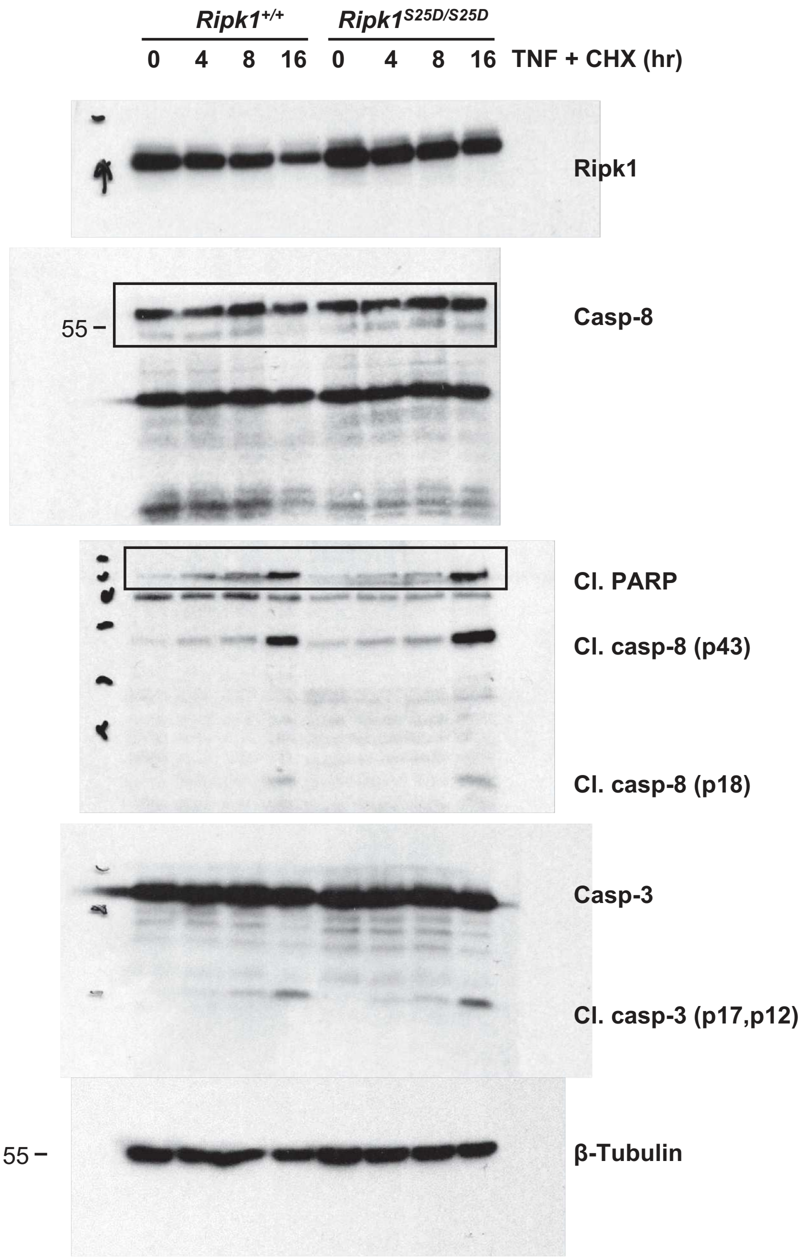

Supplementary Figure 5: Source data immunoblots (continued)

Suppl. Figure 4C

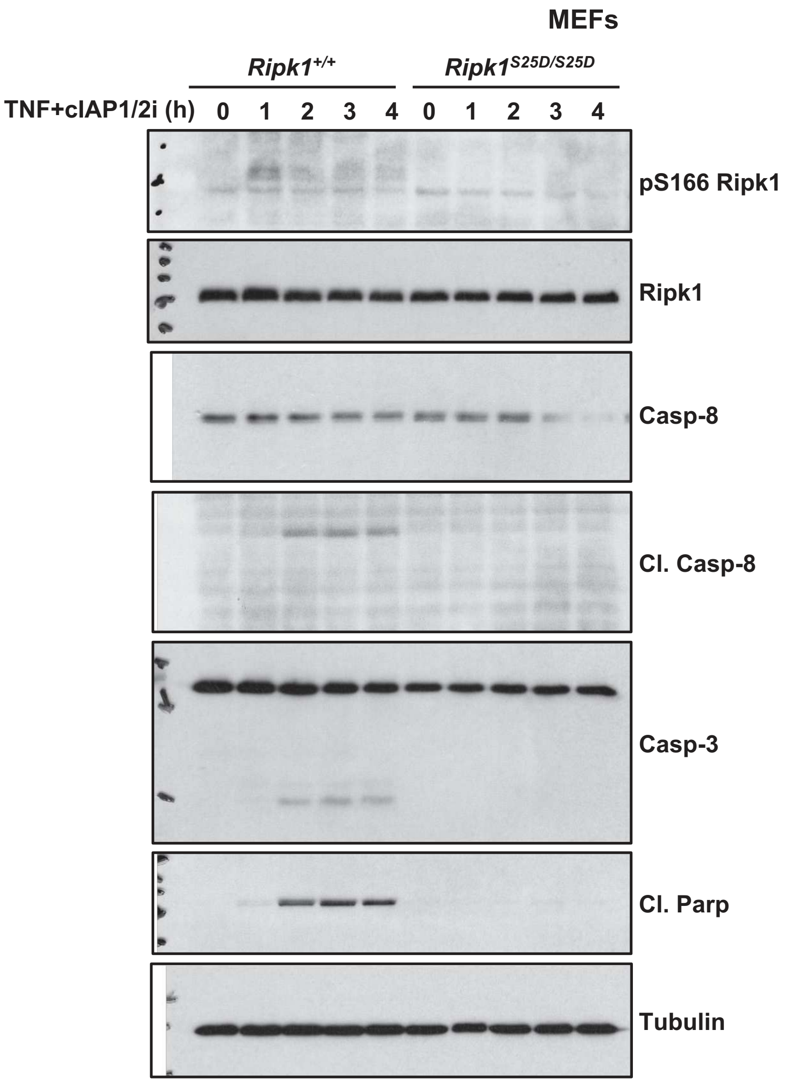

Supplementary Figure 5: Source data immunoblots (continued)

Suppl. Figure 4E

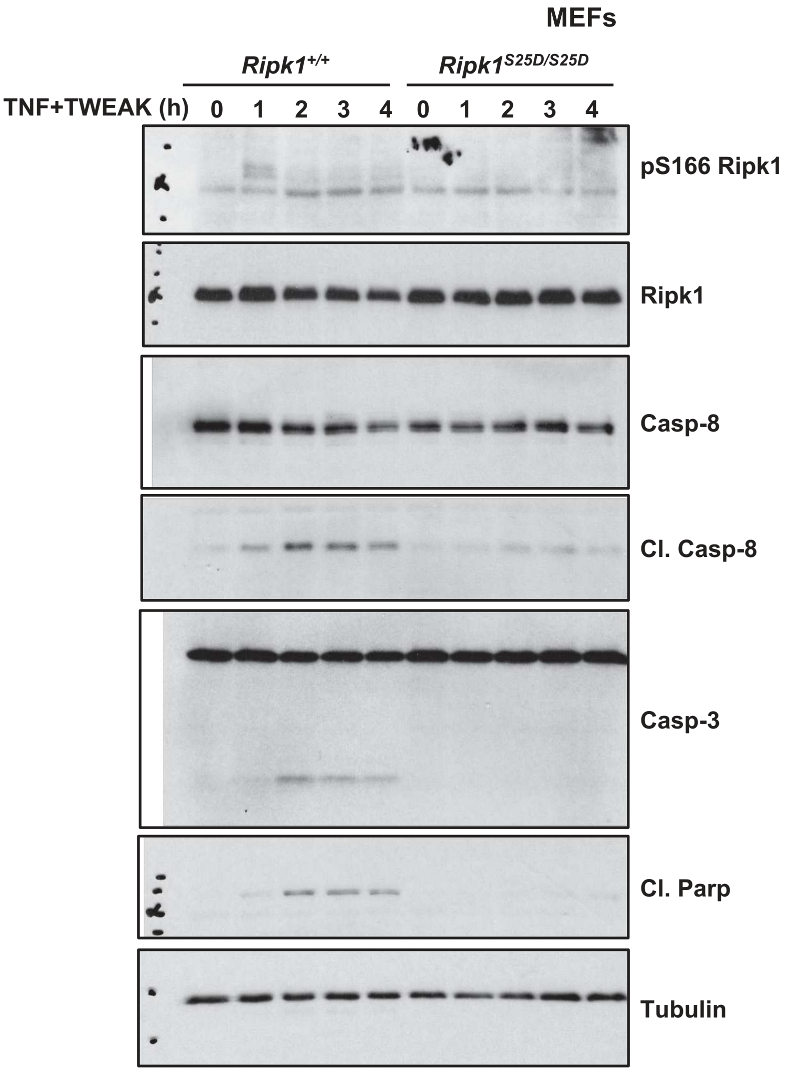

Supplementary Methods

Mass spectrometric analysis of phospho-peptides

Proteins digestion and phospho-peptide enrichment: Proteins were reduced, alkylated and digested using the FASP protocol, as described previously [1]. Digestion was performed for 4 hours with LysC (Wako) after which the mixture was diluted to 2M urea and digested with trypsin (Promega) at 37 °C overnight. Finally, the sample was acidified with formic acid to a final concentration of 5%. Tryptic peptides were desalted using Sep-Pak C18 cartridges (Waters), dried *in vacuo* and stored at -80 °C. Phospho-peptides enrichment was performed essentially as previously described [2]. Briefly, the Ti^{4+}-IMAC beads were loaded onto GELoader tips (Eppendorf) using a C8 plug and in parallel spin tip enrichment was used. The columns were conditioned using 50 µL of loading buffer consisting of 6% trifluoroacetic acid (TFA) in 80% acetonitrile (ACN). Tryptic peptides were dissolved in the loading buffer, transferred to the spin tips and centrifuged at 100 × *g* for 30 min. The columns were sequentially washed with 50 µL of 50% ACN, 0.5% TFA in 200 mM NaCl, followed by additional washing with 50 µL of 0.1% TFA in 50% ACN. The bound phospho-peptides were eluted into a new tube with 20 µL of 10% ammonia and a final elution was performed with 5 µL of 2% formic acid (FA) in 80% ACN. The collected eluate was further acidified by adding 3 µL of 100% FA, dried *in vacuo* and stored at -80 °C, until LC-MS/MS analysis.

RP nanoLC-MS/MS: The data were acquired using an UHPLC 1290 system (Agilent) coupled to an Orbitrap Q Exactive Plus mass spectrometer (Thermo Scientific). Peptides were first trapped (Dr Maisch Reprosil C18, 3 µm, 2 cm x 100 µm i.d.) before being separated on an analytical column (Agilent Poroshell EC-C18, 2.7 µm, 50 cm x 75 µm i.d.). Trapping was performed for 10 min in solvent A (0.1% FA acid in water) at 5 µL/min, and the gradient was as follows: 13-41% solvent B (0.1 % FA in 80% ACN) in 35 min, 41-100% in 3 min, and finally 100% for 1 min before returning in 1 min to 100% solvent A until the end of the run. Total analysis time was 60 min. Flow was passively split from 200 µL/min to ~300 nL/min [3]. The mass spectrometer was operated in data-dependent mode. Full-scan MS spectra from m/z 375 to m/z 1600 were acquired at a resolution of 35,000 at m/z 400, after accumulation to a target value of $3e^6$. Up to 10 most intense precursor ions were selected for fragmentation. HCD fragmentation was performed at normalized collision energy of 25 after the accumulation to a target value of $5e^4$ and a maximum injection time of 120 ms. MS/MS spectra were acquired at a resolution of 17,500. Dynamic exclusion was set to 10 s.

Data analysis: Raw data were processed with MaxQuant version 1.5.3.28 [4]. The MS/MS spectra were searched against a Swissprot Mus Musculus database version 2015_10 (16,727

entries) using the Andromeda search engine. The database search was performed with the following parameters: an initial mass tolerance of ±20 ppm and a final mass tolerance of ±4.5 ppm for precursor masses, ±20 ppm for HCD Orbitrap fragment ions, enzyme specificity was set to trypsin allowing up to two missed cleavages. Cysteine carbamidomethylation was used as a fixed modification, whereas methionine oxidation, protein N-terminal acetylation and serine, threonine and tyrosine phosphorylation as variable modifications. The false discovery rate was set to 0.01 for peptides, proteins and phosphosites, the minimum peptide length allowed was seven amino acids and a minimum Andromeda peptide score for modified peptides of 40 was required. Label-free quantification was performed, and the match between runs feature was enabled. Data analysis was performed in the Perseus computational platform [5]. A site localization probability of at least 0.75 was used as threshold for the localization of phosphoresidues.

Supplementary References

1 Wisniewski, J. R., Zougman, A., Nagaraj, N. & Mann, M. Universal sample preparation method for proteome analysis. *Nat Methods* **6**, 359-362, doi:10.1038/nmeth.1322 (2009).

2 Zhou, H. *et al.* Robust phosphoproteome enrichment using monodisperse microsphere-based immobilized titanium (IV) ion affinity chromatography. *Nat Protoc* **8**, 461-480, doi:10.1038/nprot.2013.010 (2013).

3 Cristobal, A. *et al.* In-house construction of a UHPLC system enabling the identification of over 4000 protein groups in a single analysis. *Analyst* **137**, 3541-3548, doi:10.1039/c2an35445d (2012).

4 Cox, J. & Mann, M. MaxQuant enables high peptide identification rates, individualized p.p.b.-range mass accuracies and proteome-wide protein quantification. *Nat Biotechnol* **26**, 1367-1372, doi:10.1038/nbt.1511 (2008).

5 Tyanova, S. *et al.* The Perseus computational platform for comprehensive analysis of (prote)omics data. *Nat Methods* **13**, 731-740, doi:10.1038/nmeth.3901 (2016).
