# Supplementary material for: Serine 25 phosphorylation inhibits RIPK1 kinase-dependent cell death in models of infection and inflammation
Source: Nat Commun. 2019 Apr 15;10:1729. doi: 10.1038/s41467-019-09690-0 (PMC6465317; doi:10.1038/s41467-019-09690-0)
Supplement: Supplementary file 3 — Description of Additional Supplementary Files [file 41467_2019_9690_MOESM3_ESM.pdf]

Description of Additional Supplementary Files

File Name: Supplementary Data 1
Description: Full overview of the MaxQUANT analysis of the RP-nano LC MS/MS experiment. WT MEFs and *Ikkα*$^{-/-}$ *Ikkβ*$^{-/-}$ MEFs were stimulated for 5min with 2µg/ml FLAG-hTNF before TNFR1 complex I immunoprecipitation. The phosphoproteome of the FLAG-eluted protein complexes were then compared between the two cell lines.
