# Supplementary material for: Serine 25 phosphorylation inhibits RIPK1 kinase-dependent cell death in models of infection and inflammation
Source: Nat Commun. 2019 Apr 15;10:1729. doi: 10.1038/s41467-019-09690-0 (PMC6465317; doi:10.1038/s41467-019-09690-0)
Supplement: Supplementary file 5 — Reporting Summary [file 41467_2019_9690_MOESM5_ESM.pdf]

nature research

Corresponding author(s): Prof. Dr. Mathieu Bertrand

Last updated by author(s): Mar 6, 2019

Reporting Summary

Nature Research wishes to improve the reproducibility of the work that we publish. This form provides structure for consistency and transparency in reporting. For further information on Nature Research policies, see Authors & Referees and the Editorial Policy Checklist.

Statistics

For all statistical analyses, confirm that the following items are present in the figure legend, table legend, main text, or Methods section.

n/a	Confirmed	
☐	☒	The exact sample size (*n*) for each experimental group/condition, given as a discrete number and unit of measurement
☐	☒	A statement on whether measurements were taken from distinct samples or whether the same sample was measured repeatedly
☐	☒	The statistical test(s) used AND whether they are one- or two-sided<br>*Only common tests should be described solely by name; describe more complex techniques in the Methods section.*
☒	☐	A description of all covariates tested
☐	☒	A description of any assumptions or corrections, such as tests of normality and adjustment for multiple comparisons
☐	☒	A full description of the statistical parameters including central tendency (e.g. means) or other basic estimates (e.g. regression coefficient) AND variation (e.g. standard deviation) or associated estimates of uncertainty (e.g. confidence intervals)
☐	☒	For null hypothesis testing, the test statistic (e.g. *F*, *t*, *r*) with confidence intervals, effect sizes, degrees of freedom and *P* value noted<br>*Give P values as exact values whenever suitable.*
☒	☐	For Bayesian analysis, information on the choice of priors and Markov chain Monte Carlo settings
☒	☐	For hierarchical and complex designs, identification of the appropriate level for tests and full reporting of outcomes
☒	☐	Estimates of effect sizes (e.g. Cohen's *d*, Pearson's *r*), indicating how they were calculated

Our web collection on statistics for biologists contains articles on many of the points above.

Software and code

Policy information about availability of computer code

Data collection	No software was used for data collection
Data analysis	For the in vivo experiment: sample size estimation was done by using G power 3.1.<br>Statistical analyses were performed with GraphPad Prism V7.

For manuscripts utilizing custom algorithms or software that are central to the research but not yet described in published literature, software must be made available to editors/reviewers. We strongly encourage code deposition in a community repository (e.g. GitHub). See the Nature Research guidelines for submitting code & software for further information.

Data

Policy information about availability of data

All manuscripts must include a data availability statement. This statement should provide the following information, where applicable:

- Accession codes, unique identifiers, or web links for publicly available datasets
- A list of figures that have associated raw data
- A description of any restrictions on data availability

The authors declare that the data supporting the findings of this study are available within the article and Supplementary Information Files or available from the authors upon reasonable request. Mass spectrometry data are available at https://www.ebi.ac.uk/pride/archive/login
Username: reviewer41874@ebi.ac.uk
Password: wNBtXKii

Field-specific reporting

Please select the one below that is the best fit for your research. If you are not sure, read the appropriate sections before making your selection.

☒ Life sciences ☐ Behavioural & social sciences ☐ Ecological, evolutionary & environmental sciences

For a reference copy of the document with all sections, see nature.com/documents/nr-reporting-summary-flat.pdf

Life sciences study design

All studies must disclose on these points even when the disclosure is negative.

Sample size	For the in vivo experiment: sample size estimation was done by using G power 3.1. For the in vitro cell death assays: data are represented as a mean +/- SEM of at least 3 independent experiments. Cell death assays were performed at least 3 times to allow statistical analysis
Data exclusions	No data were excluded for statistical analysis
Replication	All data in the manuscript, except for the mass spectrometry analysis, are independently repeated at least once.
Randomization	Samples were not randomized
Blinding	Investigators were not blinded to group allocation during data collection and/or analysis

Reporting for specific materials, systems and methods

We require information from authors about some types of materials, experimental systems and methods used in many studies. Here, indicate whether each material, system or method listed is relevant to your study. If you are not sure if a list item applies to your research, read the appropriate section before selecting a response.

Materials & experimental systems

n/a	Involved in the study	
☐	☒	Antibodies
☐	☒	Eukaryotic cell lines
☒	☐	Palaeontology
☐	☒	Animals and other organisms
☒	☐	Human research participants
☒	☐	Clinical data

Methods

n/a	Involved in the study	
☒	☐	ChIP-seq
☒	☐	Flow cytometry
☒	☐	MRI-based neuroimaging

Antibodies

Antibodies used	anti-RIPK1 (BD Biosciences #610459, 1:2000; Cell Signaling #3493, 1:2000), anti-pSer32/Ser36 IκBα (Cell Signaling #9246, 1:2000), anti-IκBα (Santa Cruz sc-371, 1:2000), anti-actin (MP Biomedicals #69100, 1:20000), anti-IKKα (Cell Signaling #2682, 1:1000), anti-IKKβ (Cell Signaling #2684, 1:1000), anti-β-tubulin-HRP (Abcam ab21058, 1:10000), anti-pThr180/Tyr182 p38 (Cell Signaling #9211, 1:2000), anti-p38 (Cell Signaling #9212, 1:2000), anti-pThr183/Tyr185 JNK (Invitrogen #44-682G; 1:1000), anti-JNK (Cell Signaling #9252, 1:1000), anti-pSer166 RIPK1 (Cell Signaling #31122, 1:1000), anti-cleaved caspase-8 (Cell Signaling #9429, 1:1000), anti-caspase-8 (Abnova MAB3429, 1:1000), anti-caspase-3 (Cell Signaling #9662, 1:1000), anti-cleaved PARP (Cell Signaling #9544S¸1:1000), anti-pSer345 MLKL (Millipore MABC1158, 1:1000), anti-MLKL (Millipore MABC604, 1:1000), anti-FADD (Enzo Life Sciences ADI-AAM-212-E, 1:1000), anti-SHARPIN (Proteintech 14626-1-AP, 1:2000), anti-TRADD (Bio-Rad AHP2533, 1:1000), anti-FLAG-HRP (Sigma-Aldrich A8592, 1:1000) and anti-thiophosphate ester antibody (Epitomics #2686-1).
Validation	Validation of commercial antibodies can be found at the manufacturer's website. The anti-phospho Ser25 RIPK1 antibody was validated by reconstitution of Ripk1-/- cells with phosphodeficient RIPK1 (Supplementary Figure 1) and specificity was tested at ThermoFisher Scientific

Eukaryotic cell lines

Policy information about cell lines

Cell line source(s)	Primary Ripk1+/+ and Ripk1S25D/S25D MEFs were isolated from E12.5 littermate embryos following standard protocol. MEFs were subsequently immortalized by an SV40-containing construct with JetPRIME (Polyplus transfection) according to the manufacturer’s instructions. Wild-type and Ikkα/β-/- MEFs were previously described 22,58. Ripk1+/+; Shpn+/+, Ripk1+/+; Shpncpdm/cpdm, Ripk1S25D/S25D; Shpn+/+ and Ripk1S25D/S25D; Shpncpdm/cpdm MDFs were isolated from ears of 8-10-week-old mice following standard protocol. Bone marrow-derived macrophages (BMDMs) were isolated by flushing the femurs and tibias of 7-12-week-old mice. RIPK1-/-were a kind gift of Prof. Brian Seed

Authentication	None of the cell lines were authenticated
Mycoplasma contamination	All cell lines were routinely tested for Mycoplasma contamination
Commonly misidentified lines (See ICLAC register)	No commonly misidentified cell lines were used

Animals and other organisms

Policy information about studies involving animals; ARRIVE guidelines recommended for reporting animal research

Laboratory animals	All experiments on mice were conducted according to institutional,national and European animal regulations. Animal protocols were approved by the ethics committee of Ghent University.<br>Generation of the Ripk1S25D/S25D knockin mice using the CRISPR-cas9 strategy was outsourced to Cyagen (USA). The AGC serine-encoding codon in exon 2 of the mouse RIPK1 locus was altered to a GAT codon encoding for glutamate. To do so, Cas9 mRNA, gRNA (5'-gacctagacagcggaggcttcgg-3') generated by in vitro transcription and oligo donor (with targeting sequence, flanked by 120 bp homologous sequences combined on both sides) were co-injected into fertilized C57BL/6 mouse eggs for KI mouse production. F1 heterozygous breeding pairs were obtained from Cyagen and used for establishment of the Ripk1S25D/S25D colony. Sharpincpdm mice were kindly provided by Prof. H. Walczak and were described earlier 42. Crosses between the Sharpincpdm and Ripk1S25D/S25D were validated by genotyping. All in vivo experiments were performed with littermate mice. Primary cultures were isolated from littermate mice
Wild animals	The study did not involve wild animals
Field-collected samples	No field-collected samples were used
Ethics oversight	All experiments on mice were conducted according to institutional, national and European animal regulations. Animal protocols were approved by the ethical committee of Ghent University (EC2017-071, EC2017-079)

Note that full information on the approval of the study protocol must also be provided in the manuscript.
